# Targeted Isolation of ω-3 Polyunsaturated Fatty Acids from the Marine Dinoflagellate *Prorocentrum lima* Using DeepSAT and LC-MS/MS and Their High Activity in Promoting Microglial Functions

**DOI:** 10.3390/md23070286

**Published:** 2025-07-10

**Authors:** Chang-Rong Lai, Meng-Xing Jiang, Dan-Mei Tian, Wei Lu, Bin Wu, Jin-Shan Tang, Yi Zou, Song-Hui Lv, Xin-Sheng Yao

**Affiliations:** 1 School of Traditional Chinese Materia Medica, Shenyang Pharmaceutical University, Shenyang 110016, China; laichangrong97@163.com; 2 College of Life Science and Technology, Jinan University, Guangzhou 510362, China; normanj@stu2019.jnu.edu.cn; 3 Institute of Traditional Chinese Medicine and Natural Products, College of Pharmacy/State Key Laboratory of Bioactive Molecules and Druggability Assessment/International Cooperative Laboratory of Traditional Chinese Medicine Modernization and Innovative Drug Development of Ministry of Education (MOE) of China, Jinan University, Guangzhou 510632, China; danmeitianjnu@163.com (D.-M.T.); luweilw1221@163.com (W.L.); 4 Ocean College, Zhejiang University, Zhoushan Campus, Zhoushan 316021, China; wubin@zju.edu.cn; 5 Research Center of Harmful Algae and Marine Biology, College of Life Science and Technology, Jinan University, Guangzhou 510362, China; lusonghui1963@163.com

**Keywords:** *Prorocentrum lima*, omega-3 polyunsaturated fatty acid (ω-3 PUFA), targeted isolation, DeepSAT, UPLC-MS/MS, microglia migration, amyloid-β42 clearance

## Abstract

In this study, we integrated HSQC-based DeepSAT with UPLC-MS/MS to guide the isolation of omega-3 polyunsaturated fatty acid derivatives (PUFAs) from marine resources. Through this approach, four new (**1**–**4**) and nine known (**5**–**13**) PUFA analogues were obtained from large-scale cultures of the marine dinoflagellate *Prorocentrum lima*, with lipidomic profiling identifying FA18:5 (**5**), FA18:4 (**7**), FA22:6 (**8**), and FA22:6 methyl ester (**11**) as major constituents of the algal oil extract. Structural elucidation was achieved through integrated spectroscopic analyses of IR, 1D and 2D NMR, and HR-ESI-MS data. Given the pivotal role of microglia in Alzheimer’s disease (AD) pathogenesis, we further evaluated the neuroprotective potential of these PUFAs by assessing their regulatory effects on critical microglial functions in human microglia clone 3 (HMC3) cells, including chemotactic migration and amyloid-β42 (Aβ42) phagocytic clearance. Pharmacological evaluation demonstrated that FA20:5 butanediol ester (**1**), FA18:5 (**5**), FA18:4 (**7**), FA22:6 (**8**), and (*Z*)-10-nonadecenoic acid (**13**) significantly enhanced HMC3 migration in a wound-healing assay. Notably, FA18:4 (**7**) also significantly promoted Aβ42 phagocytosis by HMC3 microglia while maintaining cellular viability and avoiding pro-inflammatory activation at 20 μM. Collectively, our study suggests that FA18:4 (**7**) modulates microglial function in vitro, indicating its potential to exert neuroprotective effects.

## 1. Introduction

Omega-3 polyunsaturated fatty acids (ω-3 PUFAs), mainly consisting of eicosapentaenoic acid (EPA, FA20:5) and docosahexaenoic acid (DHA, FA22:6), are essential bioactive lipids predominantly sourced from marine products [1]. Traditional marine organisms, fish, shrimp, and shellfish, remain primary sources of ω-3 PUFAs [2]. Recently, marine dinoflagellates have emerged as an underexplored reservoir of these bioactive lipids [3]. Notably, *Prorocentrum lima* (*P. lima*), a prolific producer of bioactive secondary metabolites, presents a compelling candidate for novel lipid discovery [4,5]. Furthermore, ω-3 PUFAs have demonstrated diverse pharmacological properties, including antioxidant, anti-inflammatory, neuroprotective, and triglyceride-lowering effects, underscoring their therapeutic potential in mitigating chronic inflammatory and neurodegenerative disorders [6,7,8,9]. Formulations of ω-3 PUFAs, containing FA20:5 (EPA) and FA22:6 (DHA), have been developed as nutraceuticals and epidemiological studies suggested that dietary supplementation with these PUFAs may confer specific neuroprotective benefits [6]. For instance, FA22:6 (DHA) reduced H_2_O_2_-induced oxidative damage via the activation of the TrkB-ERK1/2-CREB signaling pathway in cultured neural PC12 cells [7]. Additionally, enhanced phagocytic clearance of Alzheimer’s disease-associated amyloid-β42 was also observed in microglial cells by ω-3 PUFAs [9].

Recent decades have seen significant advances in preventing redundant rediscovery of known compounds and accelerating novel compound identification. The Global Natural Products Social (GNPS) molecular networking platform has emerged as a key technology by aligning mass spectrometry (MS) fragmentation spectra to enable metabolite annotation in complex mixtures [10]. We have previously successfully applied this approach in discovering novel bioactive compounds from marine fungi [10,11]. However, comprehensive chemical characterization of the petroleum ether (PE)-soluble fraction in the methanol extracts of *P. lima* remained challenging, even with molecular networking. To address this gap, DeepSAT, an innovative NMR-based structural annotation tool, has shown promise through its convolutional neural network trained on extensive spectral datasets. This system predicts molecular weights, classifies structural types, and identifies related compounds via ^1^H-^13^C HSQC correlations [12]. Complementing this, ultra-performance liquid chromatography–tandem mass spectrometry (UPLC-MS/MS) has proven to be a powerful tool for identifying compounds in complex mixtures [13]. The integration of DeepSAT’s NMR-driven structural annotation with UPLC-MS/MS-based elucidation offers a powerful strategy to accelerate novel compound discovery while improving identification workflows.

This study established an integrated DeepSAT and UPLC-MS/MS platform for systematic characterization of the PE-soluble fraction in the methanol extracts of *P. lima*. Combinatorial analysis revealed omega-3 unsaturated fatty acid esters as dominant constituents, with structural annotation identifying several novel ω-3 PUFAs. Subsequently, the PE–soluble fraction was subjected to further isolation, resulting in the isolation of four new (**1**–**4**) and nine known (**5**–**13**) PUFA analogues. In addition, the potential influences of these ω-3 PUFAs in regulating microglial activities were analyzed using the wound-healing assay and phagocytosis assay. Our results suggested that FA18:4 (**7**), an octadecatetraenoic acid, showed potential to promote microglial migration and Aβ42 phagocytosis in vitro.

## 2. Results

### 2.1. Characterization of ω-3 Polyunsaturated Fatty Esters via Integrated DeepSAT and UPLC-MS/MS

**Figure 1:**
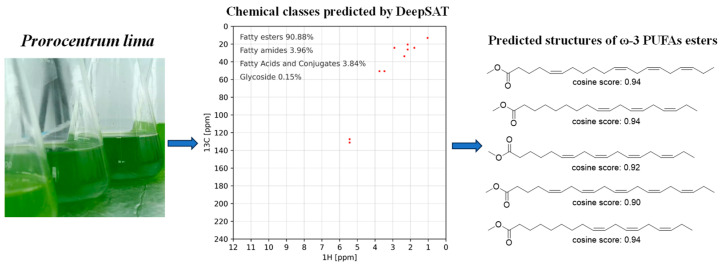
DeepSAT-based prioritization of the HSQC spectrum of petroleum ether (PE)-soluble fraction of *P. lima* and the DeepSAT results (top five structures based on cosine similarity score) for PE fraction of *P. lima*.

The methanol extracts of *P. lima* were partitioned with PE through liquid–liquid extraction using 80% aqueous methanol. Compositional analysis of the PE-soluble fraction was conducted through an integrated DeepSAT and UPLC-MS/MS workflow. For NMR-based characterization, a 30 mg aliquot was dissolved in CDCl_3_ and subjected to HSQC spectral acquisition. The processed HSQC data were then uploaded to the DeepSAT platform (https://deepsat.ucsd.edu/, accessed on 24 April 2025). As shown in Figure 1, the PE-soluble fraction mainly contained fatty acid esters (90.88%), with ω-3 PUFA esters constituting 50% of the top-ranked structural matches. This orthogonal analytical approach conclusively established the PE fraction’s chemical identity as ω-3 PUFA ester derivatives.

**Figure 2:**
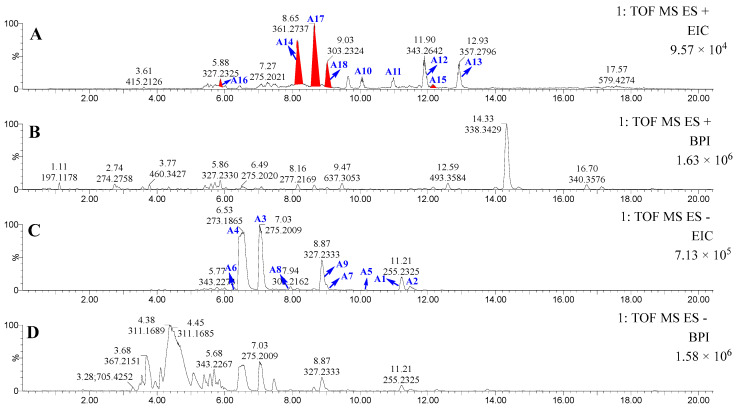
The UPLC-MS/MS results of PE-soluble fraction of *P. lima.* (**A**): Extracted ion chromatograms (EICs) in the positive-ion mode. (**B**): Base peak intensity (BPI) chromatograms in the positive-ion mode. (**C**): Extracted ion chromatograms (EICs) in the negative-ion mode. (**D**): Base peak intensity (BPI) chromatograms in the negative-ion mode.

Subsequently, lipid profiling of the PE fraction of *P. lima* was performed via UPLC-MS/MS in dual ionization modes. Database alignment using LIPID MAPS (https://www.lipidmaps.org/, accessed on 24 April 2025) identified 13 known lipids (**A1**–**A13**) through accurate molecular weight and MS/MS spectral matching (Figure 2, Table 1). Diagnostic fragment ions at *m*/*z* 259.2075 (FA18:4 ester), 285.2230 (FA20:5 ester), and 311.2375 (FA22:6 ester) enabled structural class recognition. Untargeted spectral mining leveraging these characteristic fragments revealed five novel ω-3 PUFA derivatives (**A14**–**A18**) marked by red-highlighted peaks in chromatograms (Figure 2) and asterisk-annotated in Table 1, displaying homologous fragmentation patterns to established ω-3 PUFA esters.

This integrated approach ultimately characterized 18 distinct lipid species comprising 13 known and 5 novel derivatives (Figure 2 and Table 1), promoting targeted isolation of the PE fraction.

### 2.2. Structural Elucidation

**Figure 3:**
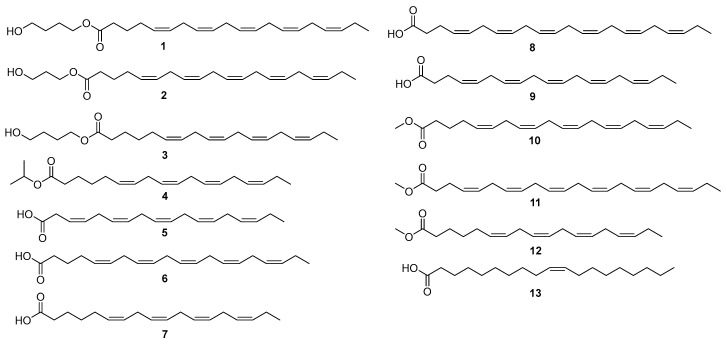
The structures of compounds **1**–**13** from the cultures of *P. lima*.

Diverse chromatographic methods were employed to isolate the chemical constituents of *P. lima*, yielding four new unsaturated fatty acids (**1**–**4**) and nine known analogues (**5**–**13**) (Figure 3). The known compounds were identified as 3,6,9,12,15-octadecapentaenoic acid (FA18:5, **5**) [14], 5,8,11,14,17-eicosapentaenoic acid (FA20:5, **6**) [15], 6,9,12,15-octadecatetraenoic acid (FA18:4, **7**) [16], 4,7,10,13,16,19-docosahexaenoic acid (FA22:6, **8**) [16], 4,7,10,13,16-nonadecanoic acid (FA19:5, **9**) [17], 5,8,11,14,17-eicosapentaenoic acid methyl ester (FA20:5 methyl ester, **10**) [18], 4,7,10,13,16,19-docosahexaenoic acid methyl ester (FA22:6 methyl ester, **11**) [16], 6,9,12,15-octadecatetraenoic acid methyl ester (FA18:4 methyl ester, **12**) [16], and (*Z*)-10-nonadecenoic acid (**13**) [19] by a comparison of their MS, ^1^H, and ^13^C NMR data with those in previous reports.

**Figure 4:**
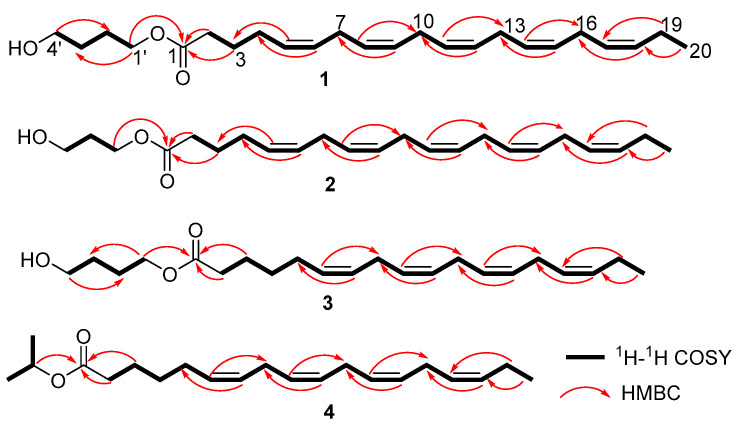
Key correlations of ^1^H-^1^H COSY and HMBC for compounds **1**–**4**.

Compound **1** was isolated as a pale-yellow oil. Its molecular formula, C_24_H_38_O_3_, was established by HR-ESI-MS ([M + H]^+^ observed at *m*/*z* 375.2897, calcd. 375.2899), suggesting six degrees of unsaturation. The IR spectrum displayed characteristic bands for a carbonyl group (1713 cm^−1^) and a hydroxyl group (3200–3400 cm^−1^). Analysis of the ^1^H and ^13^C NMR data, supported by HSQC, revealed the following key features: a carbonyl carbon at *δ*_C_ 173.8 (C-1), ten olefinic methines at *δ*_H_ 5.28–5.43 (10H, m, H-5, 6, 8, 9, 11, 12, 14, 15, 17, and 18)/*δ*_C_ 127.1–132.2 (C-5, 6, 8, 9, 11, 12, 14, 15, 17, and 18), twelve methylenes, including two oxygenated methylenes at *δ*_H_ 4.10 (2H, t, *J* = 12.8 Hz, H-1′)/*δ*_C_ 64.2 (C-1′) and *δ*_H_ 3.67 (2H, t, *J* = 12.4 Hz, H-4′)/*δ*_C_ 62.5 (C-4′), and a terminal methyl at *δ*_H_ 0.97 (3H, t, *J* = 15.2 Hz, H-20)/*δ*_C_ 14.4 (C-20). These 1D NMR features suggested compound **1** as an unsaturated fatty acid [15]. Comparative analysis with FA20:5 (**6**) indicated that they shared similar structure motifs, with main distinctions in **1** being two additional oxygenated methylenes at *δ*_H_ 4.10 (2H, t, *J* = 12.8 Hz, H-1′)/*δ*_C_ 64.2 (C-1′) and *δ*_H_ 3.67 (2H, t, H-4′, *J* = 12.4 Hz)/*δ*_C_ 62.5 (C-4′), and two methylenes at *δ*_H_ 1.75 (2H, m, H-2′)/*δ*_C_ 25.3 (C-2′) and *δ*_H_ 1.60 (2H, m, H-3′)/*δ*_C_ 29.3 (C-3′). The key ^1^H-^1^H COSY correlations of H-1′^®^H-2′^®^H-3′^®^H-4′ defined a 1′,4′-butanediol moiety in **1**, while HMBC correlations from H-1′ to C-1 anchored this moiety to the carboxyl group. Finally, the planar structure of **1** was confirmed by ^1^H-^1^H COSY and HMBC correlations (Figure 4). Furthermore, the *Z* configuration of all double bonds was assigned based on ^13^C resonances at *δ*_C_ 25.7–25.8 ppm for C-7, 10, 13, and 16 [17]. Thus, compound **1** was identified and named as (*Z*)-5,8,11,14,17-eicosapentaenoic acid 1′,4′-butanediol ester (FA20:5 butanediol ester).

Compound **2** was obtained as a pale-yellow oil. The molecular formula C_23_H_36_O_3_ was established via HR-ESI-MS ([M + H]^+^ observed at *m*/*z* 361.2743, calcd. 361.2743), corresponding to six degrees of unsaturation. The ^1^H NMR spectrum of **2** showed ten olefinic protons at *δ*_H_ 5.28–5.43 (10H, m, H-5, 6, 8, 9, 11, 12, 14, 15, 17, and 18); two oxygenated methylene protons at *δ*_H_ 4.24 (2H, t, *J* = 12.0 Hz, H-1′) and *δ*_H_ 3.69 (2H, t, *J* = 12.0 Hz, H-3′); and terminal methyl protons at *δ*_H_ 0.97 (3H, t, *J* = 15.2 Hz, H-20). The ^13^C NMR spectrum displayed 23 distinct carbon signals, including a carbonyl carbon at *δ*_C_ 174.2 (C-1), ten olefinic carbons at *δ*_C_ 127.2–132.2 (C-5, 6, 8, 9, 11, 12, 14, 15, 17, and 18), two oxygenated methylene carbons at *δ*_C_ 61.4 (C-1′) and 59.4 (C-3′), and a methyl carbon at *δ*_C_ 14.4 (C-20). Compound **2** exhibited significant structural similarity to FA20:5 (**6**) by a ^1^H and ^13^C NMR data comparison [15]. The primary distinction was the presence of two additional oxygenated methylene groups at *δ*_H_ 4.24 (2H, t, *J* = 12.0 Hz, H-1′)/*δ*_C_ 61.4 (C-1′) and *δ*_H_ 3.69 (2H, t, *J* = 12.0 Hz, H-4′)/*δ*_C_ 59.4 (C-4′) in **2**, as well as an additional methylene group at *δ*_H_ 1.87 (2H, m, H-2′)/*δ*_C_ 31.9 (C-2′). The ^1^H-^1^H COSY correlations of H-1′^®^H-2′^®^H-3′ indicated the presence of a 1′,3′-propanediol fragment in **2**, whereas the HMBC correlation between H-1′ and C-1 located the 1′,3′-propanediol moiety at C-1 in **2**. Therefore, the planar structure of **2** was determined by ^1^H-^1^H COSY and HMBC correlations (Figure 4). In addition, the ^13^C resonances of C-7, 10, 13, and 16 at *δ*_C_ 25.7–25.8 ppm suggested *Z* configurations for all double bonds [17]. Therefore, the structure of **2** was determined and named as (*Z*)-5,8,11,14,17-eicosapentaenoic acid 1′,3′-propanediol ester (FA20:5 propanediol ester).

Compound **3** was obtained as a pale-yellow oil. HR-ESI-MS showed a pseudomolecular ion at *m*/*z* 349.2746 [M + H]^+^ (calcd. 349.2743), suggesting its molecular formula as C_22_H_36_O_3_, accounting for five degrees of unsaturation. The ^1^H and ^13^C NMR data aided by the HSQC experiment revealed a carbonyl carbon at *δ*_C_ 173.9 (C-1), eight olefinic methines at *δ*_H_ 5.28–5.43 (8H, m, H-6, 7, 9, 10, 12, 13, 15, and 16)/*δ*_C_ 127.2–132.2 (C-6, 7, 9, 10, 12, 13, 15, and 16), twelve methylenes, including two oxygenated methylenes at *δ*_H_ 4.11 (2H, t, *J* = 12.8 Hz, H-1′)/*δ*_C_ 64.2 (C-1′) and *δ*_H_ 3.68 (2H, t, *J* = 12.4 Hz, H-4′)/*δ*_C_ 62.6 (C-4′), and a terminal methyl at *δ*_H_ 0.98 (3H, t, *J* = 15.2 Hz, H-18)/*δ*_C_ 14.4 (C-18). These 1D NMR characteristics indicated that **3** was an unsaturated fatty acid, similar to that of FA18:4 (**7**) [16]. The major difference between them was two additional oxygenated methylene groups at *δ*_H_ 4.11 (2H, t, *J* = 12.8 Hz, H-1′)/*δ*_C_ 64.2 (C-1′) and *δ*_H_ 3.68 (2H, t, H-4′, *J* = 12.4 Hz)/*δ*_C_ 62.6 (C-4′) and two more methylenes at *δ*_H_ 1.75 (2H, m, H-2′)/*δ*_C_ 25.3 (C-2′) and *δ*_H_ 1.60 (2H, m, H-3′)/*δ*_C_ 29.3 (C-3′) in **3**. Furthermore, the ^1^H–^1^H COSY correlations of H-1′→H-2′→H-3′→H-4′ suggested **3** has a 1′,4′-butanediol fragment, which was attached to C-1 owing to HMBC correlation between H-1′ and C-1. Finally, the planar structure of **3** was identified through the ^1^H–^1^H COSY and HMBC correlations (Figure 4). In addition, the ^13^C resonances of C-8,11,14 at *δ*_C_ 25.7–25.8 ppm identified the *Z* configurations of all double bonds [17]. Thus, the structure of **3** was determined and named as (*Z*)-6,9,12,15-octadecatetraenoic acid 1′,4′-butanediol ester (FA18:4 butanediol ester).

Compound **4** was isolated as a pale-yellow oil. Its molecular formula was assigned as C_21_H_34_O_2_ by HR-ESI-MS ([M + H]^+^ observed at *m*/*z* 319.2651, calcd. 319.2637), accounting for five degrees of unsaturation. The ^1^H and ^13^C NMR data aided by HSQC indicated that **4** was also an unsaturated fatty acid, similar to that of FA18:4 (**7**) [16]. The main difference between them was an additional oxygenated methine group at *δ*_H_ 5.03 (1H, H-1′)/*δ*_C_ 67.5 (C-1′) and two more methyl groups at *δ*_H_ 1.24 (3H, s, H-2′)/*δ*_C_ 22.0 (C-2′) and *δ*_H_ 1.26 (3H, s, H-3′)/*δ*_C_ 22.0 (C-3′) in **4**. The ^1^H–^1^H COSY correlations of H-1′→H-2′→H-3′ revealed the presence of an isopropyl alcohol fragment, which was attached to C-1 in **4** via a key HMBC correlation between H-1′ and C-1. Finally, the planar structure of **4** was determined by the ^1^H–^1^H COSY and HMBC correlations (Figure 4). The ^13^C resonances of C-8, 11, and 14 at *δ*_C_ 25.7–25.8 ppm suggested *Z* configurations of all double bonds [17]. Therefore, the structure of **4** was identified and named as (*Z*)-6,9,12,15-octadecatetraenoic acid isopropyl alcohol ester (FA18:4 isopropyl alcohol ester).

The content of isolated compounds was quantified per gram wet weight of algal cells using HPLC with an external standard one-point method. Analysis demonstrated four major constituents in the algal oil extract, with FA18:5 (**5**) 336.0 μg/g, FA18:4 (**7**) 268.0 μg/g, FA22:6 (**8**) 108.0 μg/g, and FA22:6 methyl ester (**11**) 106.8 μg/g. Other compounds were detected at lower levels (14.4–69.2 μg/g), as detailed in Table S1.

### 2.3. Biological Activity

Microglia are CNS resident innate immune cells that perform immune surveillance to maintain brain homeostasis [20]. Altered microglia functions in inflammatory responses, neurotrophic effects, and phagocytosis have been shown to associate with neuronal dysfunction, as well as with neurodegenerative disorders, such as Alzheimer’s disease [21]. The migration of activated microglia and phagocytosis of amyloid-β (Aβ) deposits play an integral role in the pathological progression of Alzheimer’s disease [22]. Unlike the well-characterized anti-inflammatory properties, the potential influence of PUFAs on microglial migration/phagocytosis remains largely unknown.

**Figure 5:**
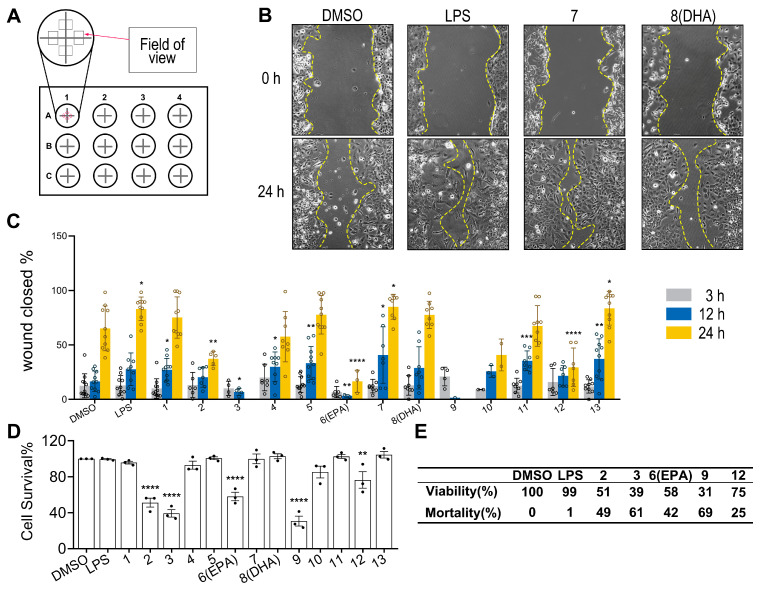
Effects of isolated compounds on HMC3 microglial motility and viability. (**A**): Schematic diagram of scratch wound healing assay: scratch regions in 12-well plates and observation time points. (**B**): Cell migration microscopy images: yellow dashed lines demarcate scratch boundaries. (**C**): Scratch healing quantification: percentage of healed area at different time points across treatment groups (*n* = 5). (**D**): Cell viability assay: HMC3 cells were treated with DMSO, LPS (1000 ng/mL), or compounds **1**–**13** (20 μM) for 24 h (*n* = 5). (**E**): Data represent the mean values of cell viability (relative to DMSO) and mortality (calculated as 100%—viability) for DMSO (control), LPS (1000 ng/mL), and FA20:5 propanediol ester (**2**), FA18:4 butanediol ester (**3**), FA20:5 (**6**, EPA), FA19:5 (**9**), and FA18:4 methyl ester (**12**) (20 μM, 24 h treatment). All significant differences were analyzed versus the DMSO group. Data presented as the mean ± SD. * *p* < 0.05, ** *p* < 0.01, *** *p* < 0.001, **** *p* < 0.0001 (one-way ANOVA, followed by least-significant-difference test).

In this section, the potential modulatory effects of these isolated ω-3 PUFAs on microglia chemotactic migration and Aβ clearance were assessed in HMC3 cells (Figure 5A). As shown by the results of the wound-healing assay, FA18:4 (**7**) and (*Z*)-10-nonadecenoic acid (**13**) significantly promoted microglia migration, while FA20:5 propanediol ester (**2**), FA20:5 (**6**, EPA), and FA18:4 methyl ester (**12**) significantly inhibited microglial migration at 20 μM 24 h after the scratches (Figure 5B,C). Although no significant effect was displayed, a trend of an increase in microglia migration was noticed with treatment of FA22:6 (**8**, DHA). Significantly increased microglia migration was also observed with the treatment of 1000 ng/mL lipopolysaccharide (LPS), which was used as a control that activated microglia, as previously reported by Pan et al. [23]. In addition, FA20:5 propanediol ester (**2**), FA18:4 butanediol ester (**3**), FA20:5 (**6**, EPA), FA19:5 (**9**), and FA18:4 methyl ester (**12**) displayed strong cytotoxicity in HMC3 cells at the tested concentration, while no significant influences on cell viabilities/proliferations were observed with other compounds (Figure 5D).

**Figure 6:**
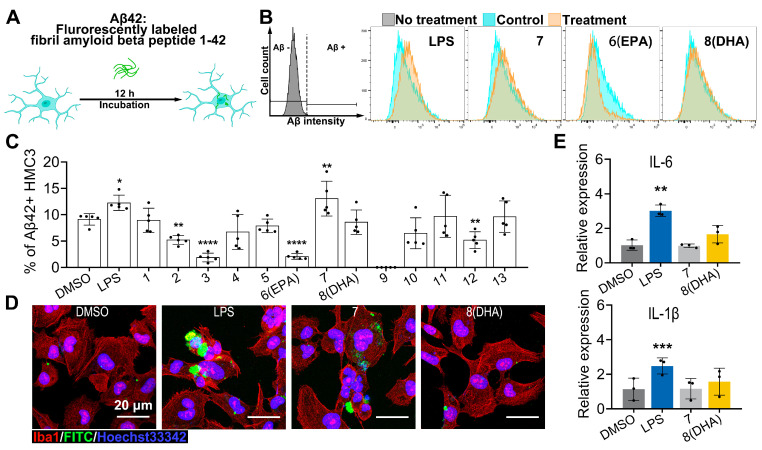
Aβ42 phagocytic clearance capacity of isolated compounds. (**A**): FITC-Aβ42 phagocytosis assay workflow: HMC3 cells co-incubated with FITC-Aβ42 for 12 h followed by flow cytometry analysis. (**B**): Representative flow cytometry plots depicting phagocytosis of FITC-Aβ42 by DAPI-positive cells in LPS (100 ng/mL), FA18:4 (**7**) (20 μM), FA20:5 (**6**, EPA, 20 μM), and FA22:6 (**8**, DHA, 20 μM)-treated groups. The fluorescence signals of cells untreated with FITC-Aβ42 were established as background (labeled as Aβ−) and served as the gating criteria for defining FITC-Aβ42 positive cells (labeled as Aβ+). (**C**): Phagocytosis rate quantification: flow cytometry detection of Aβ-positive cells (*n* = 5). (**D**): Immunofluorescence images of phagocytosis: Iba1 (red), FITC-Aβ42 (green), Hoechst (blue). Scale bar = 20 μm. (**E**): Inflammatory factor expression: qPCR analysis of *Il-6* and *Il-1β* mRNA levels (DMSO, LPS, FA18:4, and FA22:6 at 20 μM; *n* = 5). All significant differences were analyzed versus the DMSO group. Data presented as the mean ± SD. * *p* < 0.05, ** *p* < 0.01, *** *p* < 0.001, **** *p* < 0.0001 (one-way ANOVA, followed by least significance difference test).

We further assessed the potential impact of these ω-3 PUFAs on the microglial capacities of amyloid β clearance using flow cytometry cell sorting analysis of FITC-Aβ42 phagocytosis in HMC3 cells (Figure 6A). The results revealed that the treatment of 20 μM FA18:4 (**7**) significantly increased microglial Aβ42 phagocytosis, with LPS used as the positive control (Figure 6B,C). The engulfment of FITC-Aβ42 in HMC3 cells was also verified using immunofluorescence (Figure 6D).

Given the intertwined pathways of microglial activation and inflammation, the expression of pro-inflammatory cytokines upon these ω-3 PUFAs treatments was assessed using quantitative PCR. The expressions of interleukin-6 (*Il-6*) and interleukin-1β (*Il-1β*) were markedly upregulated in HMC3 cells induced with LPS, which is a well-characterized activator of macrophage inflammatory pathways. No significant induction of the expression of these pro-inflammatory mediators was observed either in HMC3 cells induced with FA18:4 (**7**) or in HMC3 cells induced with FA22:6 (**8**, DHA) (Figure 6E).

## 3. Discussion

Marine dinoflagellates represent a prolific source of bioactive natural products, with numerous compounds identified to date [24,25,26,27]. However, frequent rediscovery of known compounds hampers novel drug lead identification. To address this challenge, we developed a dual-platform workflow integrating HSQC-based DeepSAT with UPLC-MS/MS, a strategy necessitated by the suboptimal fragmentation efficiency of PUFA derivatives in conventional GNPS molecular networking. This approach enabled targeted characterization of four new (**1**–**4**) and nine known (**5**–**13**) ω-3 PUFA analogues from *P. lima*. Notably, the observed fragment ions in this study cannot be directly assigned to specific precursor ions, as individual precursors were not selected for fragmentation; the spectra represent composite fragmentation patterns under the experimental conditions used. The novelty of compounds **1**–**4**, incorporating 1′,4′-butanediol (**1**), 1′,3′-propylene glycol (**2**), 1′,4′-butanediol (**3**), and isopropanol ester (**4**) moieties, was confirmed through structural retrieval and comparison using SciFinder.

The biosynthesis of polyunsaturated fatty acids (PUFAs) in dinoflagellates is primarily mediated by an endoplasmic reticulum (ER)-associated enzyme complex comprising fatty acid desaturases (FADs) and elongases (ELOVLs) [28]. The ω-3 PUFA pathway initiates from α-linolenic acid (ALA; 18:3Δ^9,12,15^), which undergoes sequential desaturation–elongation cascades catalyzed by Δ^6^-desaturase, C18-ELOVL, Δ^5^-desaturase, and Δ^4^-desaturase, ultimately yielding long-chain ω-3 PUFAs, including FA20:5 (EPA) and FA22:6 (DHA) [28]. Subsequent enzymatic esterification of free ω-3 PUFAs with alcohols by acyltransferases and lipases produces ω-3 PUFA esters [29]. This confirms that these esters are not extraction artifacts.

Previous studies have demonstrated the neuroprotective effects of FA22:6 (DHA) at 20 μM, protecting rat pheochromocytoma PC12 cells against H_2_O_2_-induced damage and attenuating glucose deficiency-induced viability loss in murine neuronal HT-22 cells [7,30]. However, concentrations of FA22:6 (DHA) exceeding 30 μM significantly reduced HT-22 cell viability after 6 h compared with the untreated control [30]. Additionally, Abdi et al. reported that FA20:5 (EPA) and FA22:6 (DHA) induced dose-dependent death in human myeloma cell lines (L363, OPM-1, OPM-2, and U266), with pronounced effects observed at concentrations ≥ 50 μM [31]. Therefore, to mitigate potential cytotoxic effects while targeting microglial activity, we selected a moderate concentration of 20 μM for PUFAs in the present study. Our pharmacological evaluation revealed that FA18:4 (**7**) significantly enhanced both microglial migration and Aβ42 phagocytosis in vitro at 20 μM, without inducing cytotoxicity or pro-inflammatory effects. These findings support its potential as a neuroprotective agent acting through microglial regulation. FA22:6 (**8**, DHA) also displayed an increasing trend in microglia migration, although this effect did not reach statistical significance. In contrast, FA20:5 (**6**, EPA) significantly inhibited microglia migration at the same concentration (20 μM). Analysis of structure–activity relationships (SARs) revealed two critical determinants for microglial activity. First, carbon chain length appears to play a significant role. While FA18:4 (**7**) enhanced phagocytosis, longer-chain PUFAs FA20:5 (**6**, EPA) and FA22:6 (**8**, DHA) failed to stimulate phagocytosis, despite possessing anti-inflammatory properties. Second, the free carboxylic acid moiety is essential for bioactivity, as esterified analogues (FA18:4 isopropyl alcohol ester, **4**; FA18:4 methyl ester, **12**), despite sharing the same chain length as FA18:4 (**7**), lost efficacy.

Notably, we observed that FA20:5 (**6**, EPA), but not FA22:6 (**8**, DHA), exhibited significant cytotoxicity toward HMC3 microglial cells at the concentration tested. This finding contrasts with the report by Salsinha et al. [32]. This discrepancy could be attributed to potential interactions between FA20:5 (EPA) and FA22:6 (DHA), as Salsinha et al. utilized a 100 μM FA20:5 (EPA)/FA22:6 (DHA) mixture (1:1 ratio), whereas our study assessed the compounds individually. Furthermore, Abdi et al. reported that FA20:5 (EPA) (≥50 μM) induced cell death in human myeloma cell lines (L363, OPM-1, OPM-2, and U266). This variation in cytotoxic response might be explained by the inherent differences in cell type sensitivity, supported by their finding that FA20:5 (EPA) showed no cytotoxic effect on normal human peripheral blood mononuclear cells PBMCs [31].

In summary, our study establishes an integrated strategy for targeted PUFA discovery and identifies FA18:4 (**7**) as a neuroprotective lead via microglial modulation.

## 4. Materials and Methods

### 4.1. General Experimental Procedure

The IR spectra were recorded on a JASCO FT/IR-480 spectrometer (JASCO, Tokyo, Japan) and the high-resolution ESI mass spectra were acquired using a Waters Synapt G2 mass spectrometer (Waters, Wilmslow, UK). The NMR spectra were measured using a Bruker AV 400 (Bruker Co., Ltd., Bremen, Germany) with the signal of CDCl_3_ (*δ*_H_ 7.26/*δ*_C_ 77.2) as an internal reference. HPLC was performed on a Shimadzu LC-20AB and LC-20AT Liquid Chromatography, with an SPD-20A UV/VIS detector (Shimadzu, Tokyo, Japan). The analytical and semi-preparative columns included a YMC-Triart DOS column (5 μm, ϕ 4.6 mm × 250 mm) and YMC Pack ODS-A column (5 μm, ϕ 10 mm × 250 mm), respectively. Chromatographically pure MeCN and MeOH, analytically pure MeOH, petroleum ether (PE), and ethyl acetate (EtOAc) were obtained from Anaiji Chemical (Anhui Zesheng Technology, Anqing, China).

### 4.2. Culture of Prorocentrum lima

*Prorocentrum lima* was obtained from the Jinan University algae bank. It was continuously cultured in the laboratory using sterile seawater supplemented with f/2 culture medium. The culture temperature was 22 ± 1 °C, the light intensity was 45 μE m^−2^ s^−1^, the photoperiod was 12 h:12 h, and harvesting was conducted once every 30 days.

### 4.3. DeepSAT Usage Procedure

The HSQC spectrum of the PE-soluble fraction of *P. lima* was measured using a Bruker AV 400 (Bruker Co., Ltd., Bremen, Germany) with the CDCl_3_ signal (δ_H_ 7.26/δ_C_ 77.2) serving as the internal standard. The HSQC data were exported as a .csv file using MestReNova software and uploaded to the DeepSAT platform (https://deepsat.ucsd.edu/ (accessed on 24 April 2025)).

### 4.4. UPLC-MS/MS Method

The UPLC analysis was performed using an Acquity UPLC system coupled to a SYNAPTTM G2 HDMS Q-TOF tandem mass spectrometer equipped with an electrospray ionization (ESI) source (Waters, Manchester, UK). The ESI source was operated with a capillary voltage of 3 kV and a sample cone voltage of 35 V. The source temperature was maintained at 100 °C, with the desolvation gas temperature set to 300 °C. The cone gas flow rate and desolvation gas flow rate were 50 L/h and 800 L/h, respectively. Data acquisition was performed in the MSE mode. Chromatographic separation was achieved using an Acquity UPLC BEH C18 column (2.1 mm × 100 mm, 1.7 μm) with the mobile phases of eluent A (0.1% HCOOH in H_2_O, *V*/*V*) and eluent B (0.1% HCOOH in MeCN, *V*/*V*). The linear gradient program was as follows: 40–70% B (0–5 min); 70–80% B (5–9 min); 80–100% B (9–18 min); and 100% B (18–20 min). The flow rate was 0.35 mL·min^−1^, and the injection volume was 2 μL.

### 4.5. HPLC Method

The HPLC analysis was performed using a Shimadzu liquid-phase system. Chromatographic separation was achieved using an YMC C18 reverse-phase column (4.6 mm × 250 mm, 5 μm) with the mobile phases of eluent A (0.1% HCOOH in H_2_O, *V*/*V*) and eluent B (0.1% HCOOH in MeCN, *V*/*V*). The linear gradient program was as follows: 40–70% B (0–10 min); 70–100% B (10–25 min); and 100% B (25–40 min). The flow rate was 1 mL·min^−1^, the injection volume was 10 μL, and the detection wavelength was 210 nm.

### 4.6. SciFinder Search Procedure

Structural novelty was assessed using SciFinder (American Chemical Society). The complete chemical structure of each isolated compound, including atom connectivity and stereochemistry, was drawn in SciFinder’s Structure Editor and queried against the CAS Registry Database using the Exact Structure search. When the search returned no results, the compound was considered novel. Conversely, if results were obtained, the compound was identified as a known substance, as evidenced by its assigned CAS Registry Number and associated references documenting its prior synthesis or isolation.

### 4.7. Extraction and Isolation

The harvested cells were extracted with MeOH and the filtrate was concentrated to dryness under reduced pressure to obtain a crude extract (10.2 g). The crude extract was dissolved in 80% MeOH-H_2_O and partitioned into petroleum ether (PE). The PE layer (6.01 g) was subjected to silica gel column chromatography, eluting stepwise with PE-EtOAc (100:0, 98:2, 95:5, 9:1, 8:2, 7:3, 6:4, and 0:100) to obtain 5 fractions (Fr.1–5) based on HPLC and TLC analyses. Fr.2 (480 mg, PE-EtOAc 98:2 and 95:5) was divided into twelve subfractions (Fr.2–1~Fr.2–12) by Sephadex LH-20 column chromatography, eluted with MeOH. Fr.2–6 (106.5 mg) was isolated using semi-preparative HPLC (0.1% HCOOH in 90% MeOH-H_2_O) to obtain compounds **5** (8.1 mg, *t*_R_ = 9.89 min), **6** (13.7 mg, *t*_R_ = 11.54 min), and **7** (12.8 mg, *t*_R_ = 14.28 min). Fr.2–12 (158.5 mg) was isolated using semi-preparative HPLC (0.1% HCOOH in 78% MeCN-H_2_O) to obtain compounds **3** (3.5 mg, *t*_R_ = 18.15 min), **10** (24.2 mg, *t*_R_ = 21.92 min), and **11** (34.4mg, *t*_R_ = 28.11 min). Fr.3 (1.5 g, PE-EtOAc 9:1 and 8:2) was separated by Sephadex LH-20 column chromatography, and eluted with MeOH to acquire ten subfractions (Fr.3–1–Fr.3–10). Compounds **4** (3.7 mg, *t*_R_ = 13.99 min) and **8** (42.1 mg, *t*_R_ = 11.44 min) were obtained from Fr.3–3 (129.4 mg) by semipreparative HPLC eluted with 80% MeCN-H_2_O (0.1% HCOOH). Compounds **1** (46.6 mg, *t*_R_ = 9.85 min) and **9** (10.6 mg, *t*_R_ = 14.16 min) were acquired from Fr.3–6 (88.1 mg) by semipreparative chromatography eluted with 75% MeCN-H_2_O (0.1% HCOOH). Compounds **2** (4.1 mg, *t*_R_ = 11.86 min), **12** (11.6 mg, *t*_R_ = 13.92 min), and **13** (18.1 mg, *t*_R_ = 17.19 min) were isolated from Fr.3–8 (142.5 mg) using semipreparative HPLC eluted with 85% MeOH-H_2_O (0.1% HCOOH).

### 4.8. Spectroscopic Data of New Compounds

(Z)-5,8,11,14,17-eicosapentaenoic acid 1′,4′-butanediol ester (**1**): pale-yellow oil; IR (KBr) νmax: 3434, 2965, 2927, 2866, 1681, 1617, 1553, 1455, 1379, 1316, and 1220 cm^−1^; HR-ESI-MS: *m*/*z* 375.2897 [M + H]^+^ (calcd for C_24_H_38_O_3_, 375.2899); ^1^H and ^13^C NMR spectral data (Table 2).

(Z)-5,8,11,14,17-eicosapentaenoic acid 1′,3′-propanediol ester (**2**): pale-yellow oil; IR (KBr) νmax: 3427, 2936, 2864, 1684, 1568, 1410, and 1277 cm^−1^; HR-ESI-MS: *m*/*z* 361.2743 [M + H]^+^ (calcd for C_23_H_36_O_3_, 361.2743); ^1^H and ^13^C NMR spectral data (Table 2).

(Z)-6,9,12,15-octadecatetraenoic acid 1′,4′-butanediol ester (**3**): pale-yellow oil; IR (KBr) νmax: 3477, 3235, 2927, 1710, 1634, 1515, 1463, 1376, and 1261 cm^−1^; HR-ESI-MS: *m*/*z* 349.2746 [M + H]^+^ (calcd for C_22_H_36_O_3_, 349.2743); ^1^H and ^13^C NMR spectral data (Table 3).

(Z)-6,9,12,15-octadecatetraenoic acid isopropyl alcohol ester (**4**): pale-yellow oil; IR (KBr) νmax: 2938, 1716, 1537, 1410, and 1288 cm^−1^; HR-ESI-MS: *m*/*z* 319.2651 [M + H]^+^ (calcd for C_21_H_34_O_2_, 319.2637); ^1^H and ^13^C NMR spectral data (Table 3).

### 4.9. MTT Assay

Human microglia clone 3 (HMC3) cells were purchased from American Type Culture Collection (ATCC, Manassas, VA, USA). HMC3 cells were cultured in DMEM/F12 medium containing 10% FBS until reaching logarithmic growth phase, then seeded into 96-well plates at 5000 cells/well. After 24 h of pre-culture under 37 °C with 5% CO_2_, cells were treated with cell-free medium (blank control), 1000 ng/mL LPS, or 20 μM test compounds (**1**–**13**). Following 24 h treatment, 20 μL of 5 mg/mL MTT solution was added to each well and incubated for 4 h. The supernatant was discarded, and 150 μL DMSO was added to dissolve formazan crystals with agitation. Optical density (OD) was measured at 570 nm using a microplate reader. Cell viability (%) = [(OD treatment − OD blank)/(OD control − OD blank)] × 100. Each group contained 5 technical replicates, with 3 independent experiments.

### 4.10. Scratch Wound Healing Assay

HMC3 cells were seeded into 12-well culture plates and maintained in DMEM/F12 medium containing 10% FBS until reaching 95% confluency. A confluent monolayer was then scratched using a sterile pipette tip, followed by three washes with PBS to remove detached cells. The cells were subsequently cultured in medium supplemented with either DMSO (blank control), 1000 μg/mL LPS, or 20 μM compounds. Images were acquired at baseline (t = 0 h) and post-scratch time points (t = 3, 12, and 24 h) using a Motif inverted microscope equipped with Image-Q digital imaging at 10× magnification. The migration area was analyzed with Image J software (Version 1.54p). The percentage of wound closure was calculated using the following formula: wound closed (%) = (scratch area t = 0 − scratch area t = n)/scratch area t = 0 × 100, where n represents the test time point.

### 4.11. Aβ42 Peptide Phagocytosis Assay and Flow Cytometry

The FITC-conjugated Aβ142 peptide (Cat. No. M212900, MREDA, Beijing, China) was dissolved in DMSO to prepare a 200 μM stock solution stored at −80 °C. Twelve hours prior to experimentation, the stock was diluted with DMEM to generate 1 μM FITC-Aβ42 working solution, followed by 37 °C incubation to induce fibrillar aggregation. HMC3 cells were then cultured in medium containing compounds **1**–**13** along with Aβ42. Post-phagocytosis assay, cells were stained with fixable LiveDead viability dye (Thermo Fisher Scientific, Waltham, MA, USA). Aβ42 uptake was quantified via flow cytometry by detecting FITC fluorescence within microglia.

### 4.12. Reverse Transcription and Quantitative Real Time-PCR (qRT-PCR)

Total RNAs were extracted from cultured cells and tissues using TRIzol Reagent (Thermo Fisher). The concentrations and purities of RNAs were determined using a NanoDrop 1000 Spectrophotometer (Thermo Scientific, Waltham, MA, USA). The extracted RNA was reverse-transcribed to cDNA using a PrimeScript RT Reagent Kit with gDNA Eraser (cat. no. RR047A, Takara, Kusatsu, Japan) according to the manufacturer’s instructions. Quantitative Real-Time Polymerase Chain Reaction (qRT-PCR) was performed using TB Green Premix Ex TaqⅡ (Tli RNaseH Plus) (cat. no. RR820A, listed in Table 4). The quantification of target gene mRNA expression was performed using the 2^−ΔΔCt^ method and normalized to glyceraldehyde-3-phosphate dehydrogenase (GAPDH).

## Figures and Tables

**Table 1:** Fatty acyl composition of *P. lima* by UPLC-MS/MS analysis.

No.	Lipid Type (C:N)	*t*_R_ (min)	Formula	Ion Type	Calculated Ion *m*/*z*	Observed Ion *m*/*z*	Mass Accuracy Error (ppm)	Possible MS/MS Fragments
**A1**	FA16:0	11.21	C_16_H_32_O_2_	[M − H]^−^	255.2324	255.2325	0.4	239.2011
**A2**	FA18:1	11.47	C_18_H_34_O_2_	[M − H]^−^	281.2481	281.2482	0.4	265.2168
**A3**	FA18:4	7.03	C_18_H_28_O_2_	[M − H]^−^	275.2011	275.2010	−0.4	231.2113
**A4**	FA18:5	6.28	C_18_H_26_O_2_	[M − H]^−^	273.1855	273.1865	3.7	229.1958
**A5**	FA19:1	10.16	C_19_H_36_O_2_	[M − H]^−^	295.2637	295.2645	2.7	279.2321
**A6**	FA19:5	6.98	C_19_H_28_O_2_	[M − H]^−^	287.2011	287.2017	2.1	243.2113
**A7**	FA20:3	9.00	C_20_H_34_O_2_	[M − H]^−^	305.2481	305.2492	3.6	261.2582
**A8**	FA20:5	7.94	C_20_H_30_O_2_	[M − H]^−^	301.2168	301.2166	−0.7	257.2310
**A9**	FA22:6	8.87	C_22_H_32_O_2_	[M − H]^−^	327.2324	327.2333	2.8	283.2430
**A10**	FA18:4 methyl ester	10.05	C_19_H_30_O_2_	[M + H]^+^	291.2324	291.2326	0.7	277.2188, 259.2075
**A11**	FA20:5 methyl ester	10.99	C_21_H_32_O_2_	[M + H]^+^	317.2481	317.2491	3.2	303.2322, 285.2230
**A12**	FA22:6 methyl ester	11.90	C_23_H_34_O_2_	[M + H]^+^	343.2637	343.2640	0.9	329.2458, 311.2375
**A13**	FA22:6 ethyl ester	12.91	C_24_H_36_O_2_	[M + H]^+^	357.2794	357.2792	−0.6	329.2467, 311.2376
**A14** *	FA18:4 ester	8.14	C_22_H_36_O_3_	[M + H]^+^	349.2743	349.2745	0.8	277.2171, 259.2068
**A15** *	FA18:4 ester	12.15	C_21_H_34_O_2_	[M + H]^+^	319.2637	319.2630	−2.2	277.2159, 259.2058
**A16** *	FA20:5 ester	5.88	C_22_H_30_O_2_	[M + H]^+^	327.2324	327.2325	0.3	303.2325, 285.2246
**A17** *	FA20:5 ester	8.65	C_23_H_36_O_3_	[M + H]^+^	361.2743	361.2737	−0.6	303.2322, 285.2216
**A18** *	FA20:5 ester	9.03	C_24_H_38_O_3_	[M + H]^+^	375.2899	375.2899	−0.0	303.2322, 285.2216

C:N: represent the ratio of C to double bonds; * new fatty acid derivatives.

**Table 2:** Assignments of the 1D NMR signals for compounds **1** and **2** (400 MHz for ^1^H and 100 MHz for ^13^C in CDCl_3_).

No.	1		2	
	*δ*_C_	*δ*_H_ (*J* in Hz)	*δ*_C_	*δ*_H_ (*J* in Hz)
1	173.8		174.2	
2	33.9	2.31 (2H, t, 15.2)	33.8	2.32 (2H, t, 15.2)
3	25.0	1.69 (2H, m)	25.0	1.69 (2H, m)
4	26.7	2.12 (2H, m)	26.7	2.12 (2H, m)
5, 6, 8, 9, 11, 12, 14, 15, 17, 18	127.1–132.2	5.28–5.43 (10H, m)	127.2–132.2	5.28–5.43 (10H, m)
7, 10, 13, 16	25.7–25.8	2.79–2.85 (8H, m)	25.7–25.8	2.79–2.86 (8H, m)
19	20.7	2.05 (2H, m)	20.7	2.05 (2H, m)
20	14.4	0.97 (3H, t, 15.2)	14.4	0.97 (3H, t, 15.2)
1′	64.2	4.10 (2H, t, 12.8)	61.4	4.24 (2H, t, 12.0)
2′	25.3	1.75 (2H, m)	31.9	1.87 (2H, m)
3′	29.3	1.60 (2H, m)	59.4	3.69 (2H, t, 12.0)
4′	62.5	3.67 (2H, t, 12.4)		

**Table 3:** Assignments of the 1D NMR signals for compounds **3** and **4** (400 MHz for ^1^H and 100 MHz for ^13^C in CDCl_3_).

No.	3		4	
	*δ*_C_	*δ*_H_ (*J* in Hz)	*δ*_C_	*δ*_H_ (*J* in Hz)
1	173.9		173.4	
2	34.4	2.31 (2H, t, 14.8)	34.7	2.31 (2H, t, 15.2)
3	24.8	1.66 (2H, m)	24.8	1.66 (2H, m)
4	29.3	1.39 (2H, m)	29.2	1.42 (2H, m)
5	27.0	2.09 (2H, m)	27.0	2.12 (2H, m)
6, 7, 9, 10, 12, 13, 15, 16	127.2–132.2	5.28–5.43 (8H, m)	127.2–132.2	5.31–5.46 (8H, m)
8, 11, 14	25.7–25.8	2.83 (6H, m)	25.7–25.8	2.86 (6H, m)
17	20.7	2.05 (2H, m)	20.7	2.08 (2H, m)
18	14.4	0.98 (3H, t, 15.2)	14.4	1.00 (3H, t, 15.2)
1′	64.2	4.11 (2H, t, 12.8)	67.5	5.03 (1H, m)
2′	25.3	1.75 (2H, m)	22.0	1.24 (3H, s)
3′	29.3	1.60 (2H, m)	22.0	1.26 (3H, s)
4′	62.6	3.68 (2H, t, 12.4)		

**Table 4:** Primers sequence for qRT-PCR.

Gene	Sequences (5′-3′)
*Il-1β*	F: ATGATGGCTTATTACAGTGGCAA R: GTCGGAGATTCGTAGCTGGA
*Il-6*	F: TCCTTCTCCACAAACATGTAACAA R: TCACCAGGCAAGTCTCCTCA
GAPDH	F: ACACCCACTCCTCCACCTTTG R: TCCACCACCCTGTTGCTGTAG

## Data Availability

The data given in this research are available in this article and the Supplementary Materials.

## Supplementary Materials

The following supporting information can be downloaded at: https://www.mdpi.com/article/10.3390/md23070286/s1, Table S1: Content of compounds **1**–**13** per gram of wet weight of *P. lima* cells measured by HPLC; Figure S1 HSQC spectrum of petroleum ether soluble fraction of *P. lima*; Figure S2 HPLC chromatograms of the n-hexane-soluble and petroleum ether-soluble (methanol extracts) fractions of *P. lima*, along with isolated compounds **3**, **10**–**12**; Figure S3 The HPLC results of PE-soluble fraction of *P. lima* and compounds **1**–**13**; Figures S4–S62: Spectrum of compounds **1**–**13**.
