# Supplementary material for: Targeted Isolation of ω-3 Polyunsaturated Fatty Acids from the Marine Dinoflagellate Prorocentrum lima Using DeepSAT and LC-MS/MS and Their High Activity in Promoting Microglial Functions
Source: Mar Drugs. 2025 Jul 10;23(7):286. doi: 10.3390/md23070286 (PMC12298421; doi:10.3390/md23070286)
Supplement: Supplementary file 1 [file marinedrugs-23-00286-s001.zip › marinedrugs-3682257-supplementary.pdf]

Supplementary Information

Article

Targeted Isolation of ω-3 Polyunsaturated Fatty Acids from the Marine Dinoflagellate *Prorocentrum lima* using DeepSAT and LC-MS/MS and Their High Activity in Promoting Microglial Functions

Chang-Rong Lai [1,†], Meng-Xing Jiang [2,†], Dan-Mei Tian [3,†], Wei Lu [3], Bin Wu [4], Jin-Shan Tang [3,*], Yi Zou [2,*], Song-Hui Lv [5] and Xin-Sheng Yao [1,*]

[1] School of Traditional Chinese Materia Medica, Shenyang Pharmaceutical University, Shenyang 110016, Liaoning, China; laichangrong97@163.com

[2] College of Life Science and Technology, Jinan University, Guangzhou, 510362, China; normanj@stu2019.jnu.edu.cn

[3] Institute of Traditional Chinese Medicine and Natural Products, College of Pharmacy/State Key Laboratory of Bioactive Molecules and Druggability Assessment/International Cooperative Laboratory of Traditional Chinese Medicine Modernization and Innovative Drug Development of Ministry of Education (MOE) of China, Jinan University, Guangzhou 510632, China; danmeitianjnu@163.com (D.-M.T.); luweilw1221@163.com (W.L.)

[4] Ocean College, Zhejiang University, Zhoushan Campus, Zhoushan 316021, China; wubin@zju.edu.cn

[5] Research Center of Harmful Algae and Marine Biology, College of Life Science and Technology, Jinan University, Guangzhou, 510362, China; lusonghui1963@163.com

* Correspondence: gztangjinshan@126.com (J.-S.T.); tyizou@jnu.edu.cn (Y.Z.); tyaoxs@jnu.edu.cn (X.-S.Y.)

† These authors contributed equally to this work.

Contents

Table S1 Content of compounds **1-13** per gram of wet weight of *P. lima* cells measured by HPLC. 5

Figure S1 HSQC spectrum of petroleum ether soluble fraction of *P. lima*. 5

Figure S2 HPLC chromatograms of the n-hexane-soluble and petroleum ether-soluble (methanol extracts) fractions of *P. lima*, along with isolated compounds **3**, **10-12.** 6

Figure S3 The HPLC results of PE-soluble fraction of P. lima and compounds **1-13**. 6

Figure S4 The MS spectrum (A) and fragmentation pathways (B) of compound **1**. 7

Figure S5 IR spectrum of compound **1**. 7

Figure S6 The HPLC results of compounds **1**. 8

Figure S7 ^{1}H-NMR spectrum of compound **1**. 8

Figure S8 ^{13}C-NMR spectrum of compound **1**. 9

Figure S9 HSQC spectrum of compound **1**. 9

Figure S10 ^{1}H-^{1}H COSY spectrum of compound **1**. 10

Figure S11 HMBCspectrum of compound **1**. 10

Figure S12 The MS spectrum (A) and fragmentation pathways (B) of compound **2**. 11

Figure S13 IR spectrum of compound **2**. 11

Figure S14 The HPLC results of compounds **2**. 11

Figure S15 ^{1}H-NMR spectrum of compound **2**. 12

Figure S16 ^{13}C-NMR spectrum of compound **2**. 12

Figure S17 HSQC spectrum of compound **2**. 13

Figure S18 ^{1}H-^{1}H COSY spectrum of compound **2**. 13

Figure S19 HMBC spectrum of compound **2**. 14

Figure S20 The MS spectrum (A) and fragmentation pathways (B) of compound **3**. 14

Figure S21 IR spectrum of compound **3**. 15

Figure S22 The HPLC results of compounds **3**. 15

Figure S23 ^{1}H-NMR spectrum of compound **3**. 16

Figure S24 ^{13}C-NMR spectrum of compound **3**. 16

Figure S25 HSQC spectrum of compound **3**. 17

Figure S26 ^{1}H-^{1}H COSY spectrum of compound **3**. 17

Figure S27 HMBC spectrum of compound **3**. 18

Figure S28 The MS spectrum (A) and fragmentation pathways (B) of compound **4**. 18

Figure S29 IR spectrum of compound **4**. 19
Figure S30 The HPLC results of compounds **4**. 19
Figure S31 ^{1}H-NMR spectrum of compound **4**. 20
Figure S32 ^{13}C-NMR spectrum of compound **4**. 20
Figure S33 HSQC spectrum of compound **4**. 21
Figure S34 ^{1}H-^{1}H COSY spectrum of compound **4**. 21
Figure S35 HMBC spectrum of compound **4**. 22
Figure S36 The HPLC results of compounds **5**. 22
Figure S37 ^{1}H-NMR spectrum of compound **5**. 23
Figure S38 ^{13}C-NMR spectrum of compound **5**. 23
Figure S39 The HPLC results of compounds **6**. 24
Figure S40 ^{1}H-NMR spectrum of compound **6**. 24
Figure S41 ^{13}C-NMR spectrum of compound **6**. 25
Figure S42 The HPLC results of compounds **7**. 25
Figure S43 ^{1}H-NMR spectrum of compound **7**. 26
Figure S44 ^{13}C-NMR spectrum of compound **7**. 26
Figure S45 The HPLC results of compounds **8**. 27
Figure S46 ^{1}H-NMR spectrum of compound **8**. 27
Figure S47 ^{13}C-NMR spectrum of compound **8**. 28
Figure S48 The HPLC results of compounds **9**. 28
Figure S49 ^{1}H-NMR spectrum of compound **9**. 29
Figure S50 ^{13}C-NMR spectrum of compound **9**. 29
Figure S51 The HPLC results of compounds **10**. 30
Figure S52 ^{1}H-NMR spectrum of compound **10**. 30
Figure S53 ^{13}C-NMR spectrum of compound **10**. 31
Figure S54 The HPLC results of compounds **11**. 31
Figure S55 ^{1}H-NMR spectrum of compound **11**. 32
Figure S56 ^{13}C-NMR spectrum of compound **11**. 32
Figure S57 The HPLC results of compounds **12**. 33
Figure S58 ^{1}H-NMR spectrum of compound **12**. 33
Figure S59 ^{13}C-NMR spectrum of compound **12**. 34
Figure S60 The HPLC results of compounds **13**. 34

Figure S61 ^{1}H-NMR spectrum of compound **13**. 35

Figure S62 ^{13}C-NMR spectrum of compound **13**. 35

Table S1 Content of compounds **1-13** per gram of wet weight of *P. lima* cells measured by HPLC.

No.	Content (μg/g)	No.	Content (μg/g)
compound **1**	14.4	compound **8**	108.0
compound **2**	20.8	compound **9**	56.8
compound **3**	52.0	compound **10**	48.0
compound **4**	19.2	compound **11**	106.8
compound **5**	336.0	compound **12**	57.6
compound **6**	40.0	compound **13**	69.2
compound **7**	268.0		

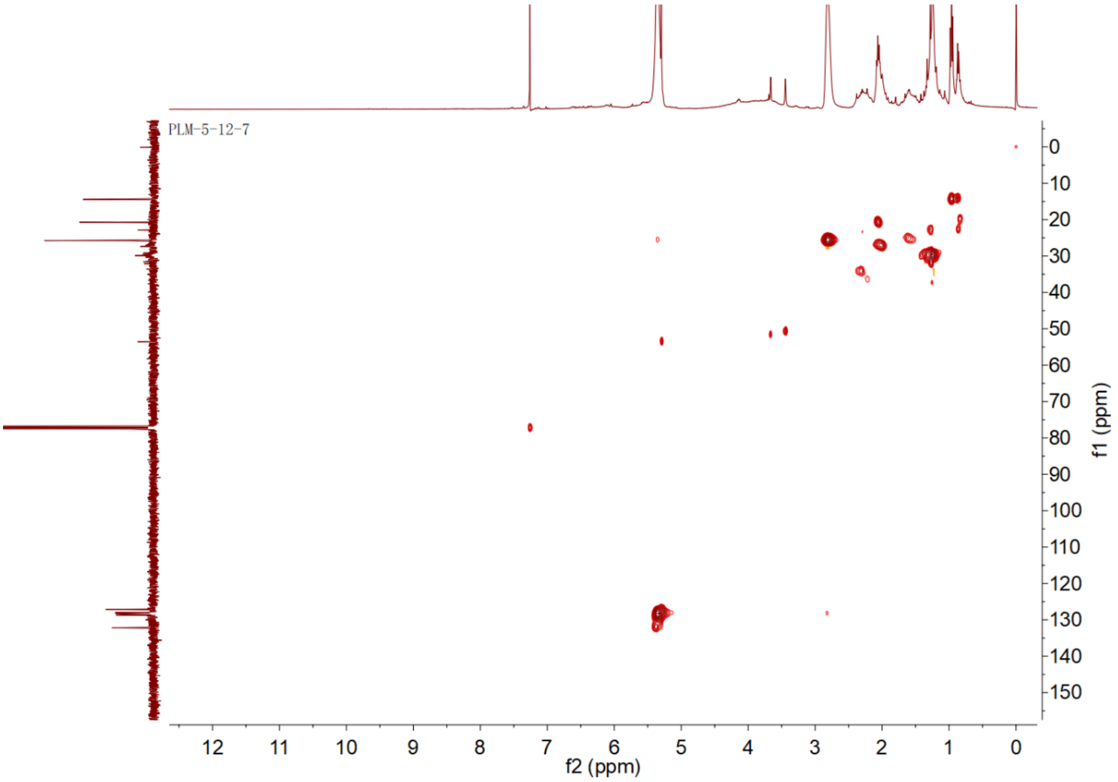

Figure S1 HSQC spectrum of petroleum ether soluble fraction of *P. lima*.

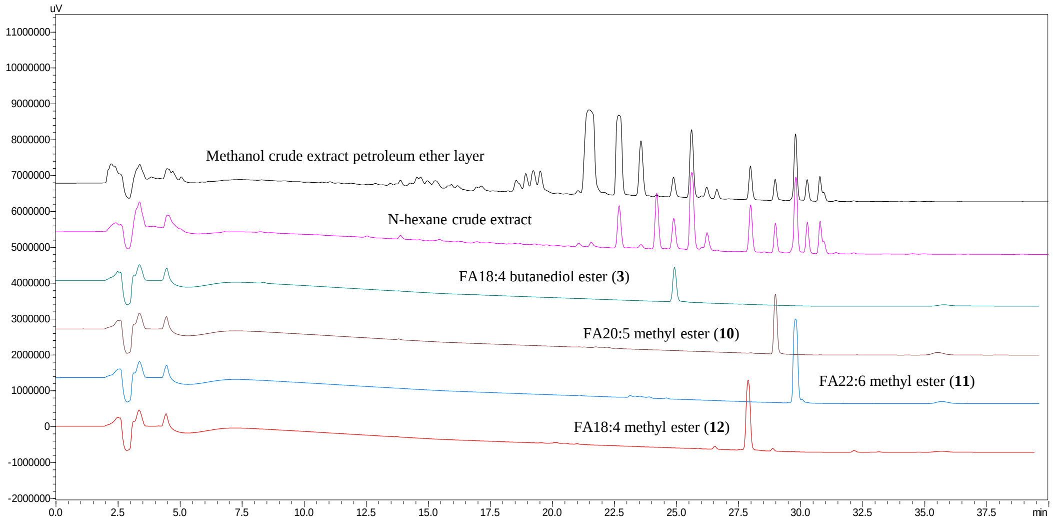

Figure S2 HPLC chromatograms of the n-hexane-soluble and petroleum ether-soluble (methanol extracts) fractions of *P. lima*, along with isolated compounds **3**, **10-12.**

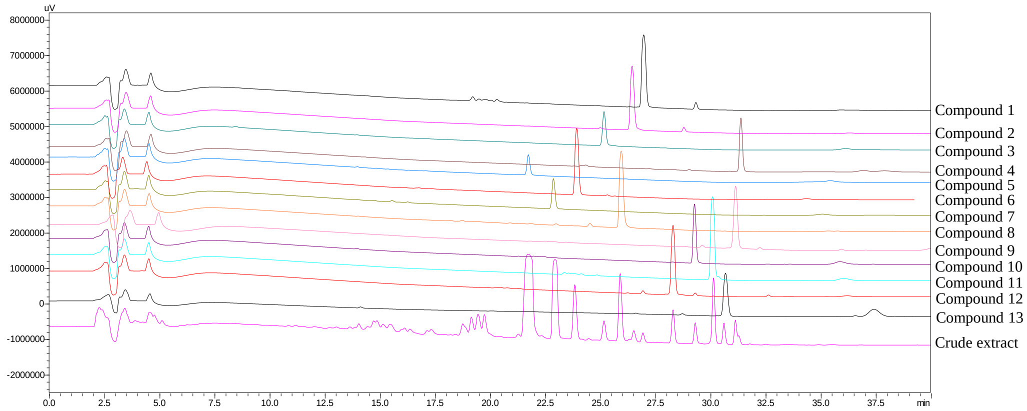

Figure S3 The HPLC results of PE-soluble fraction of P. lima and compounds **1-13**.

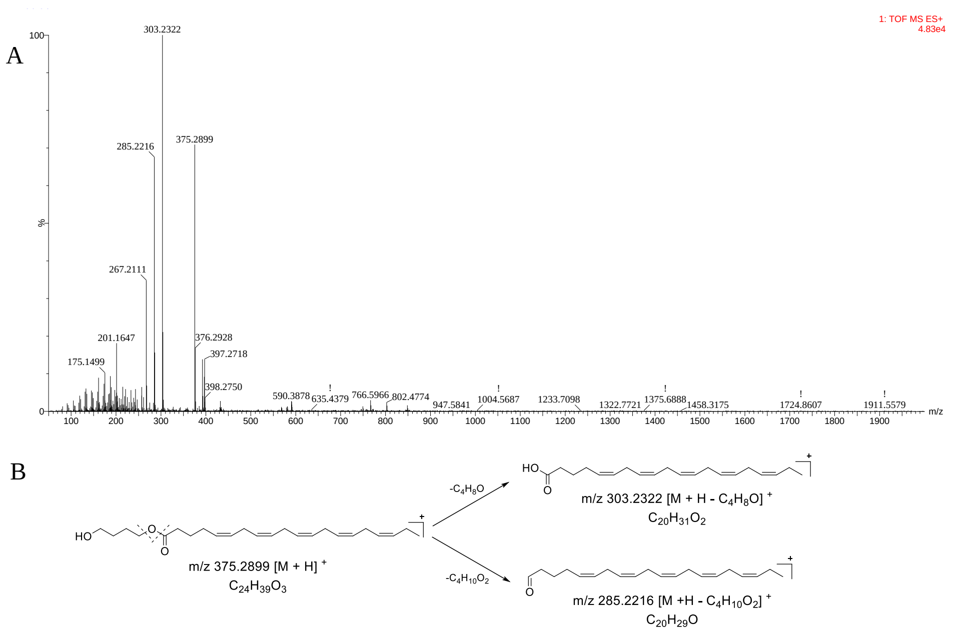

Figure S4 The MS spectrum (A) and fragmentation pathways (B) of compound **1**.

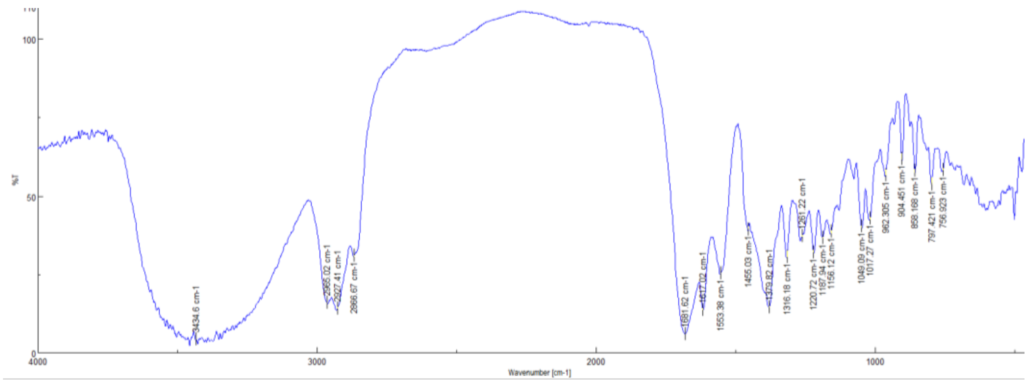

Figure S5 IR spectrum of compound **1**.

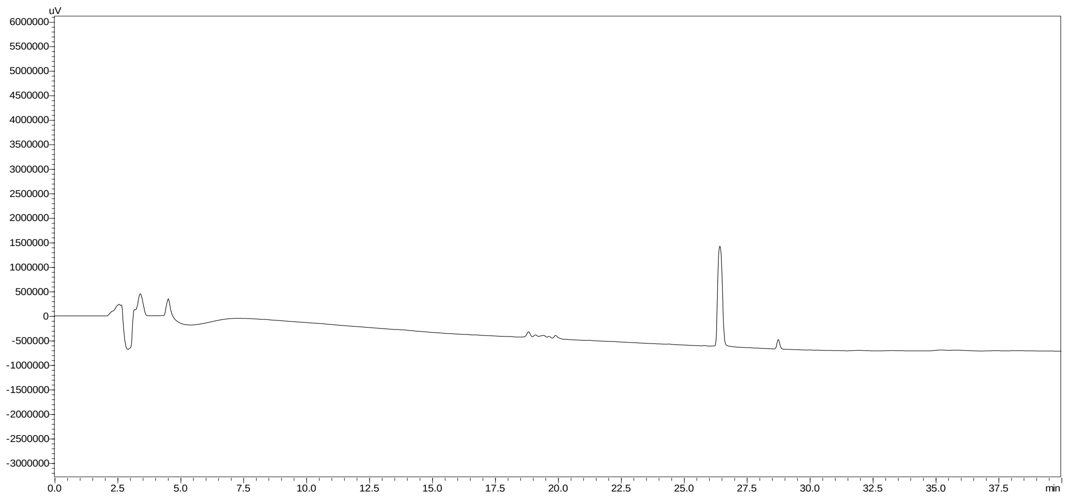

Figure S6 The HPLC results of compounds **1**.

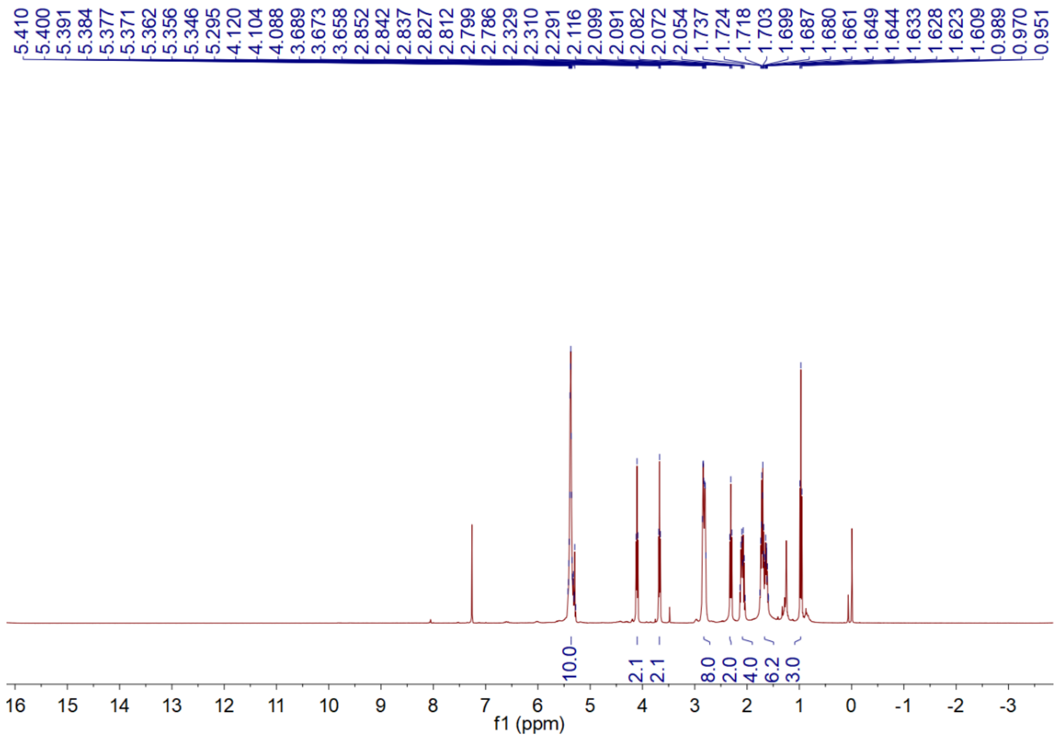

Figure S7 ^{1}H-NMR spectrum of compound **1**.

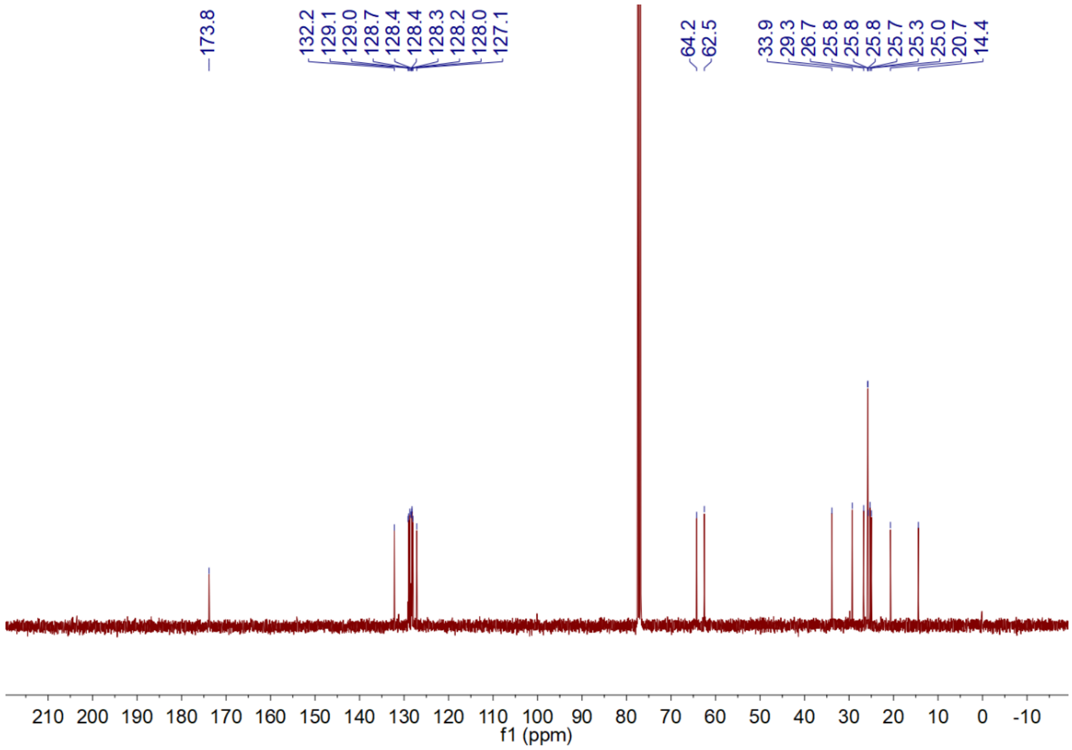

Figure S8 ^{13}C-NMR spectrum of compound **1**.

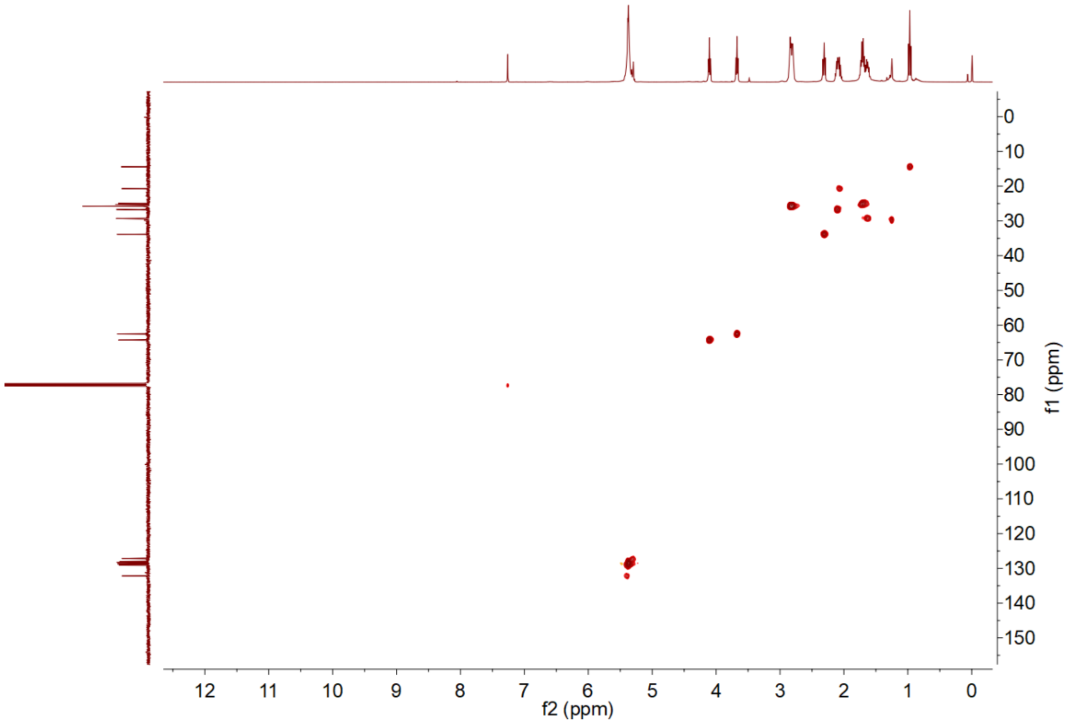

Figure S9 HSQC spectrum of compound **1**.

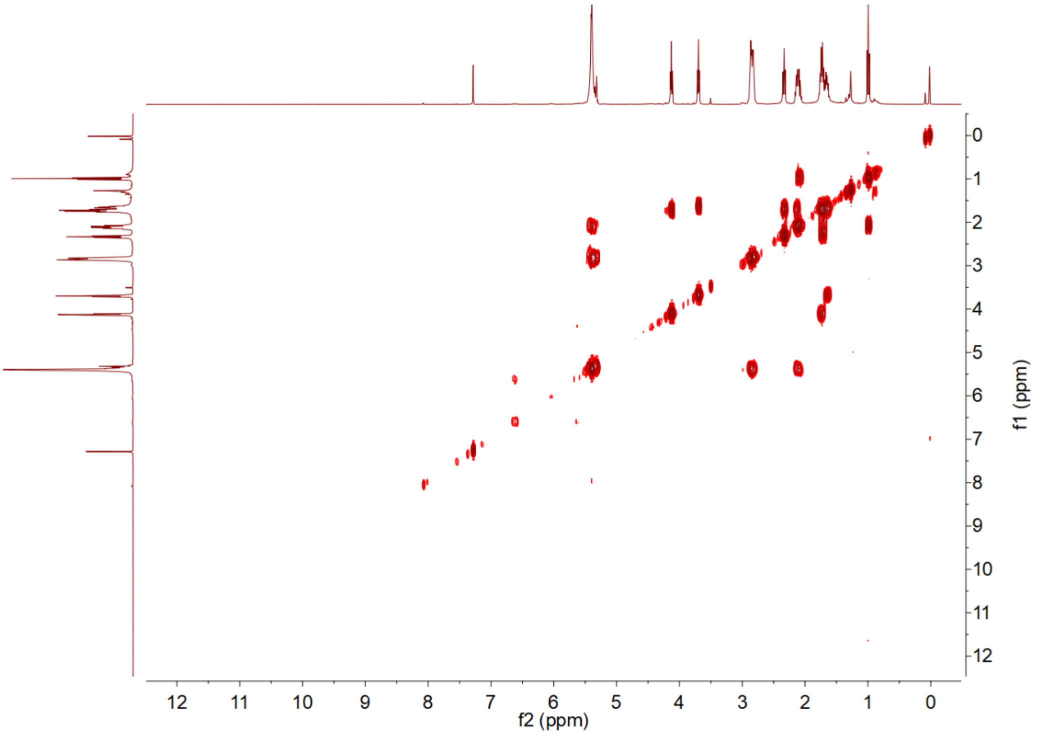

Figure S10 ^{1}H-^{1}H COSY spectrum of compound **1**.

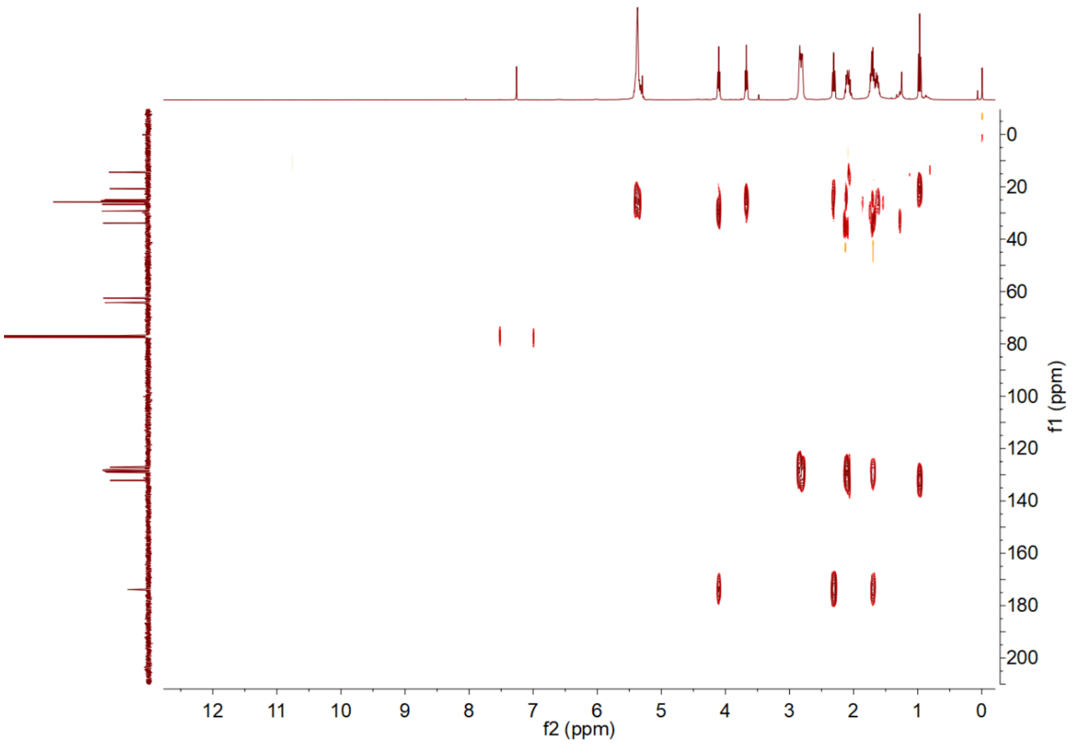

Figure S11 HMBCspectrum of compound **1**.

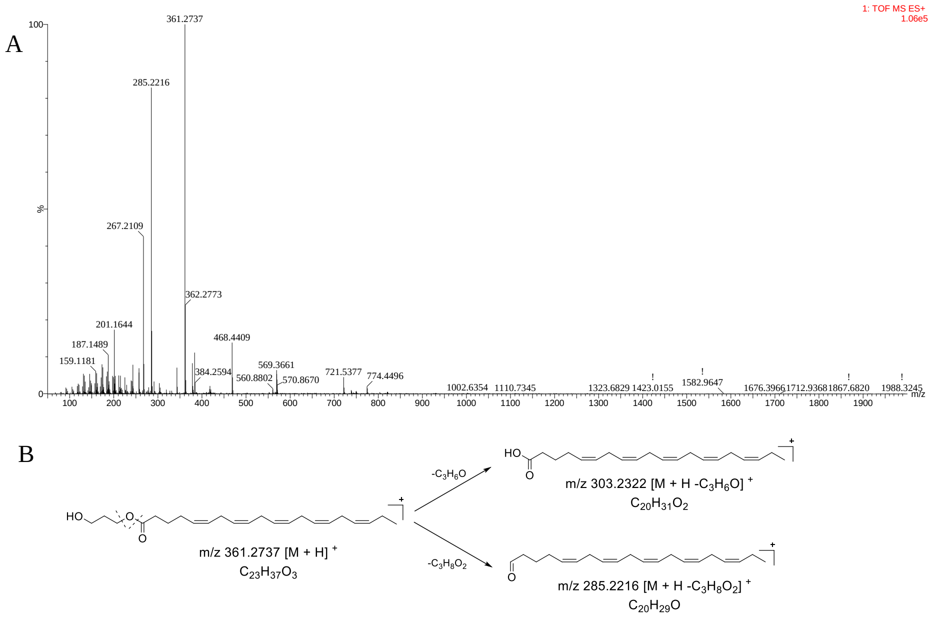

Figure S12 The MS spectrum (A) and fragmentation pathways (B) of compound **2**.

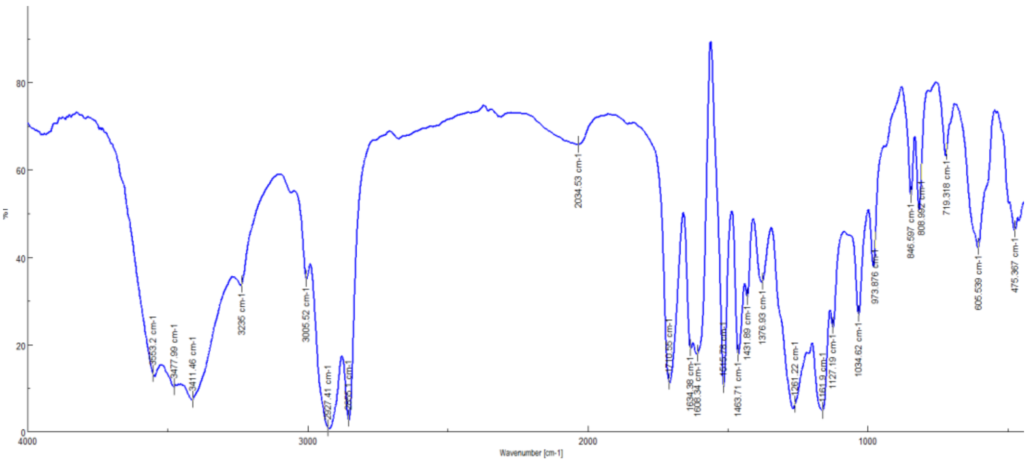

Figure S13 IR spectrum of compound **2**.

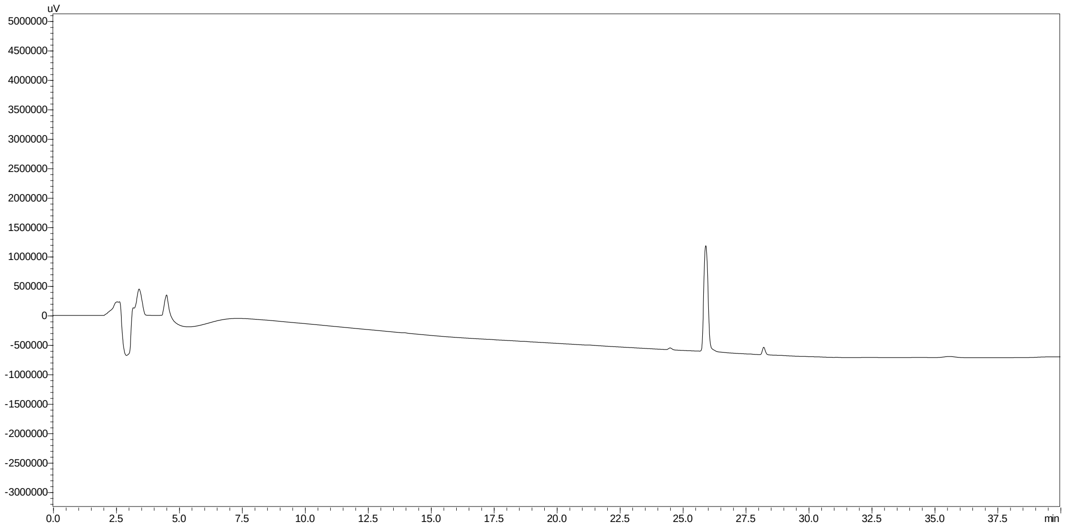

Figure S14 The HPLC results of compounds **2**.

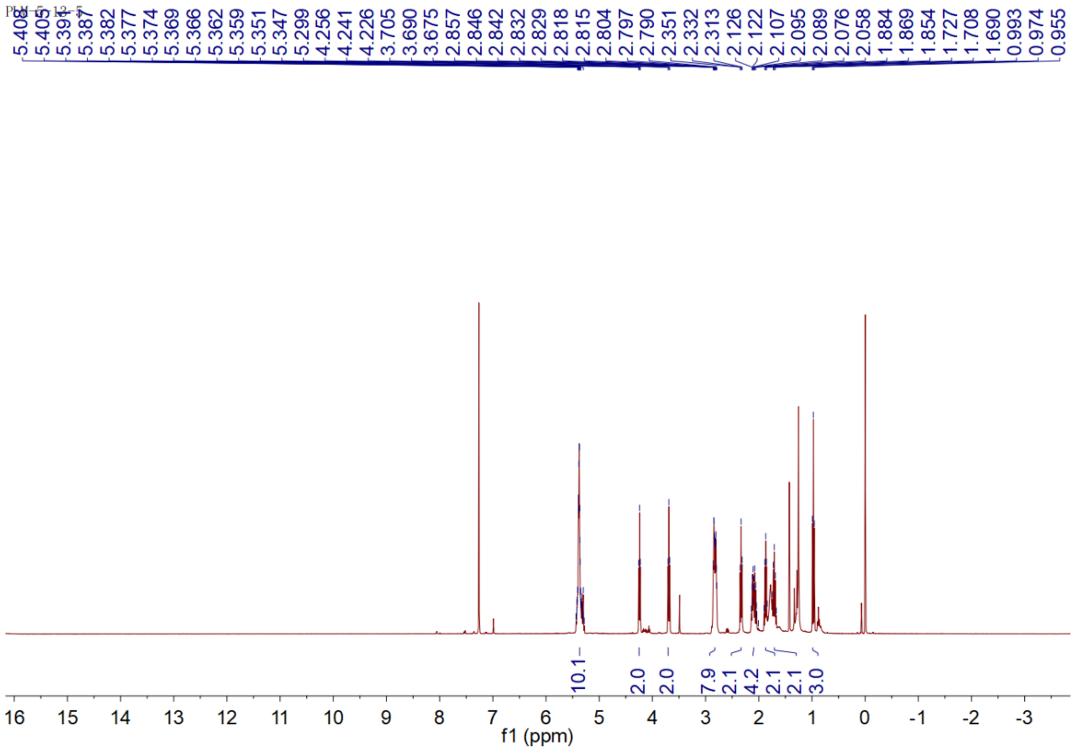

Figure S15 ^{1}H-NMR spectrum of compound **2**.

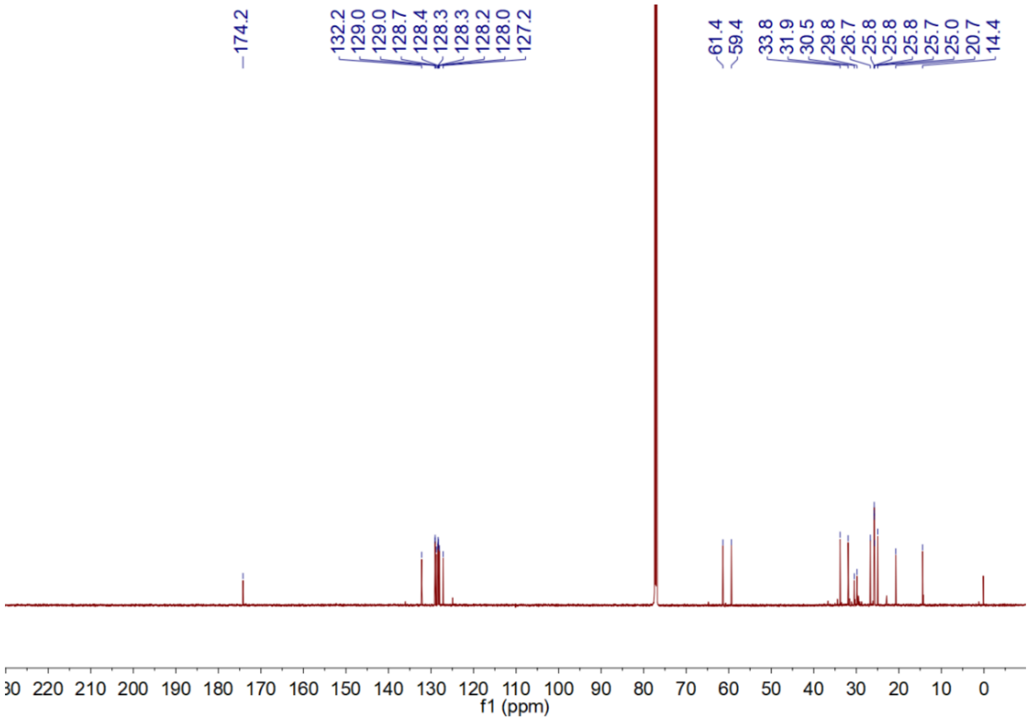

Figure S16 ^{13}C-NMR spectrum of compound **2**.

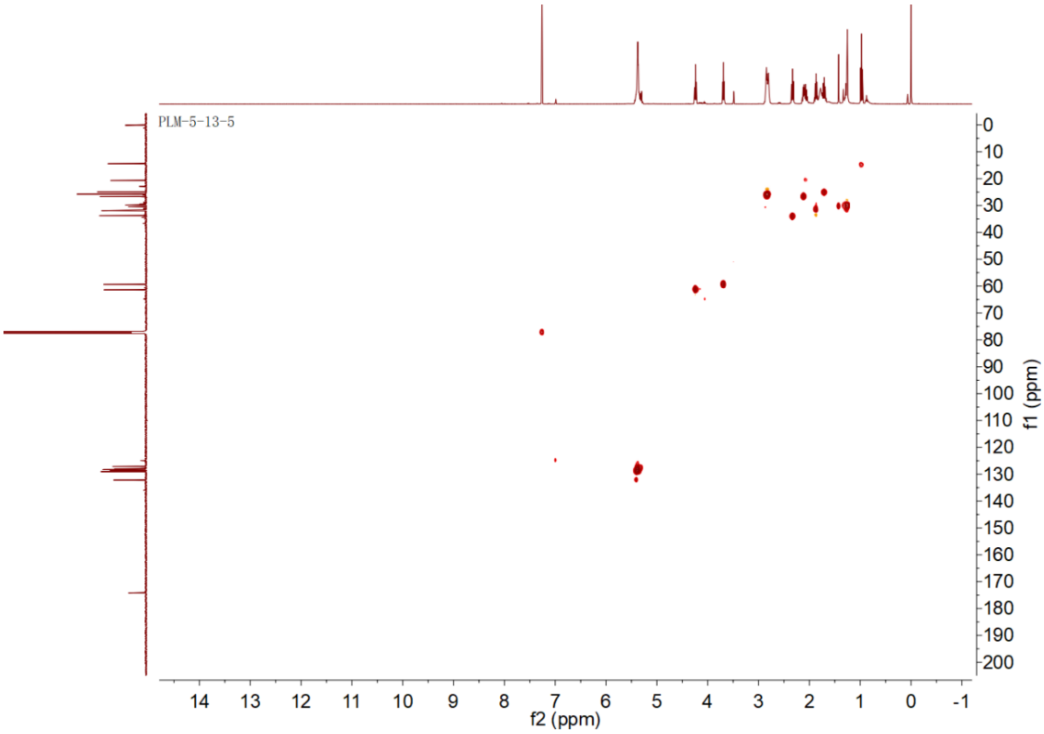

Figure S17 HSQC spectrum of compound **2**.

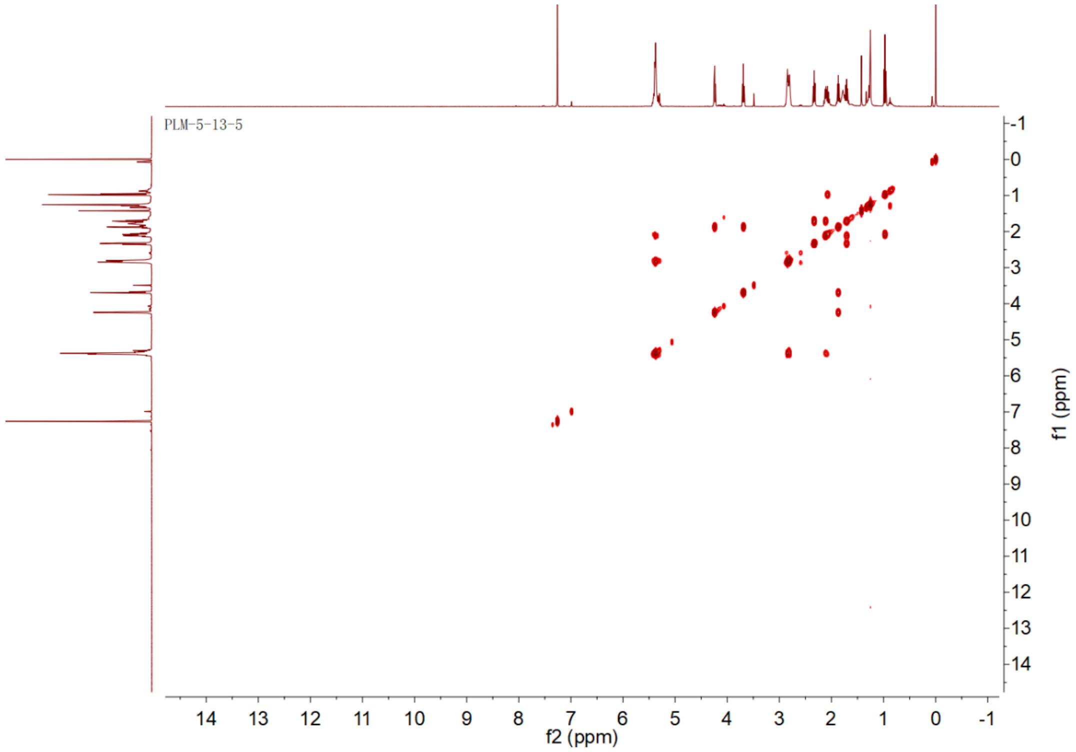

Figure S18 ^{1}H-^{1}H COSY spectrum of compound **2**.

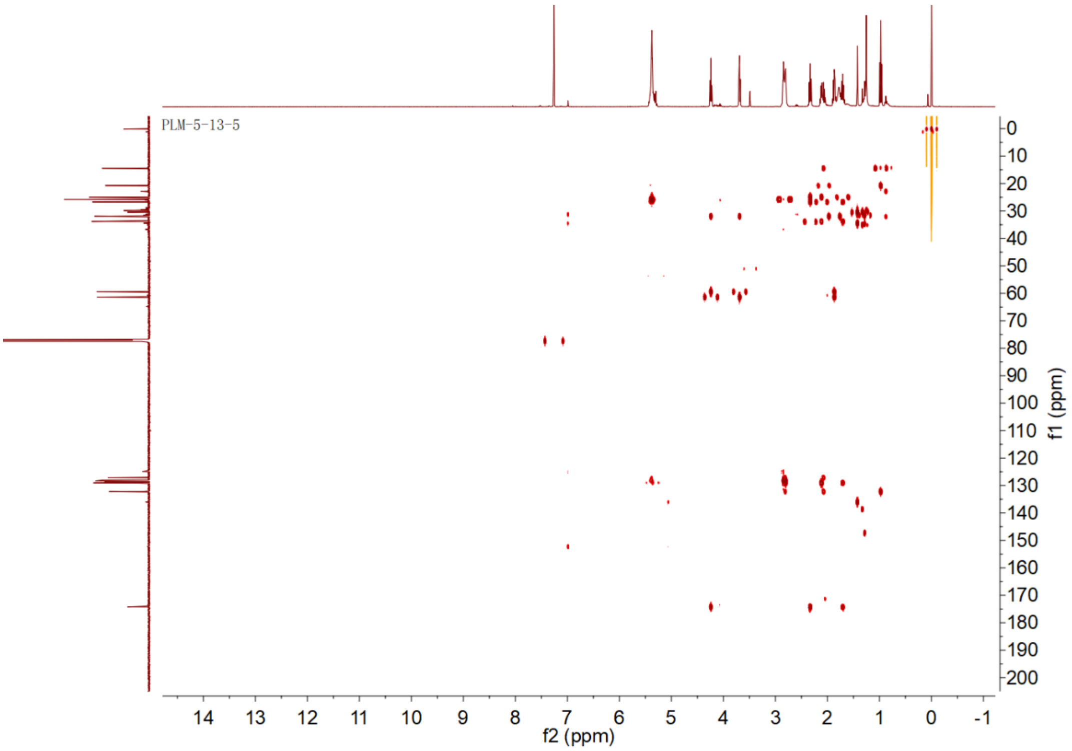

Figure S19 HMBC spectrum of compound **2**.

A

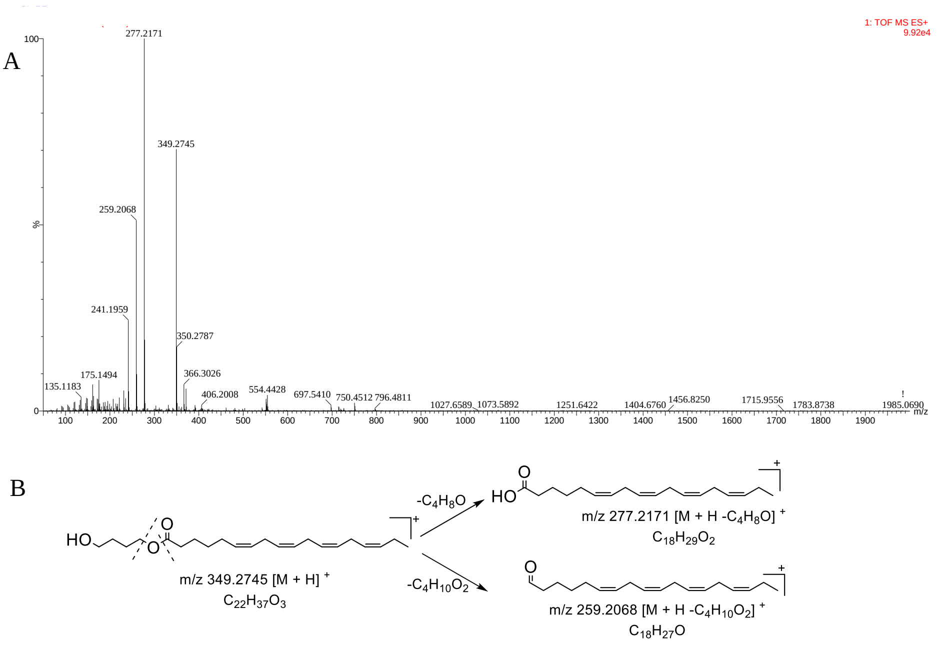

B

Figure S20 The MS spectrum (A) and fragmentation pathways (B) of compound **3**.

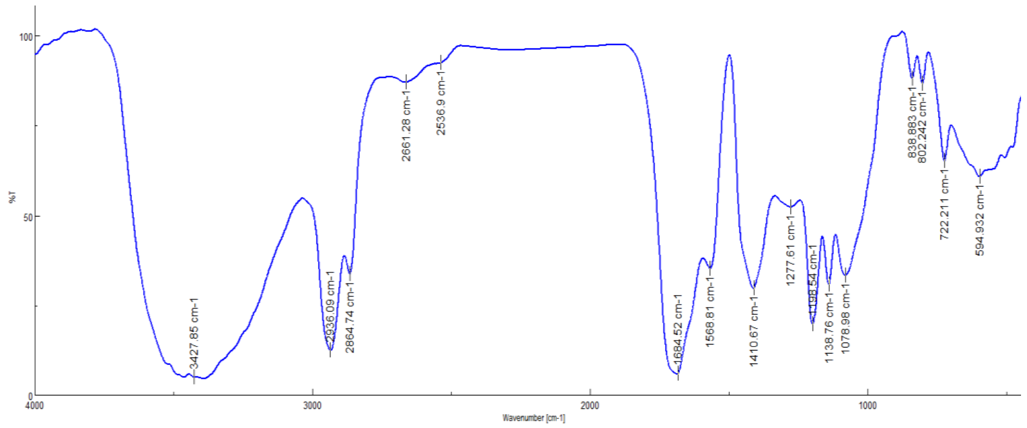

Figure S21 IR spectrum of compound **3**.

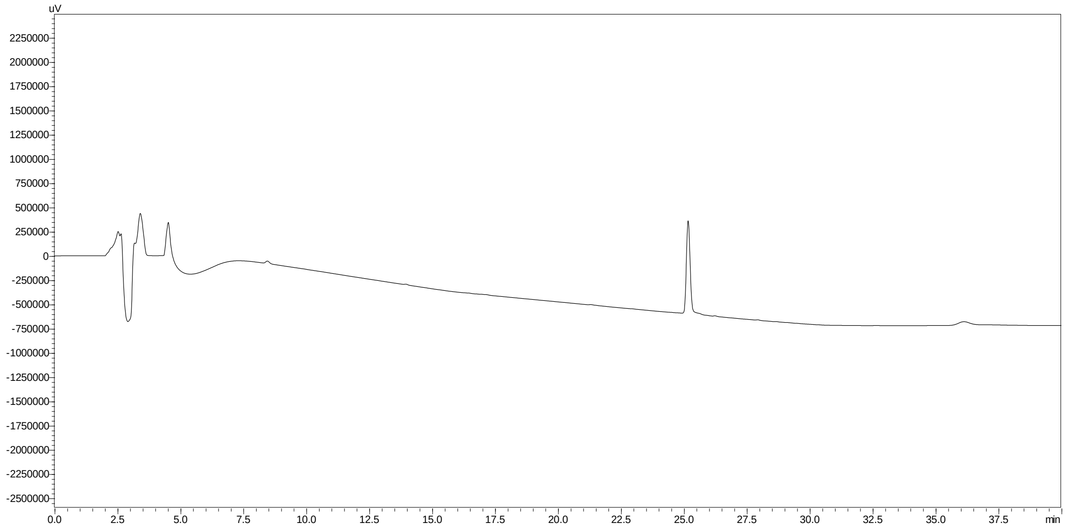

Figure S22 The HPLC results of compounds **3**.

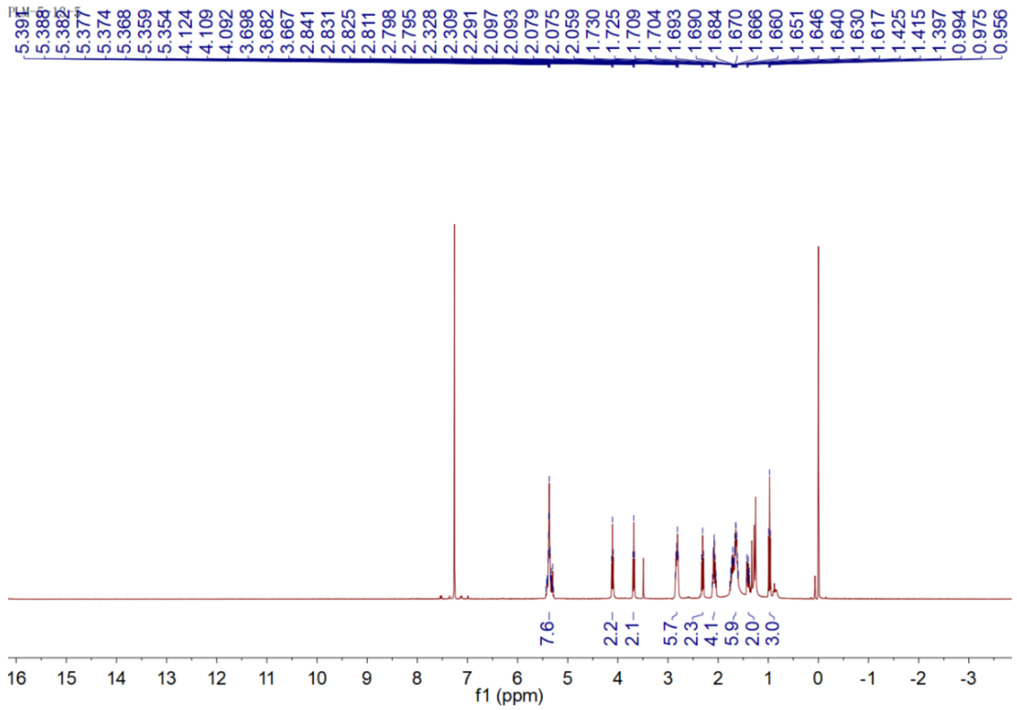

Figure S23 ^{1}H-NMR spectrum of compound **3**.

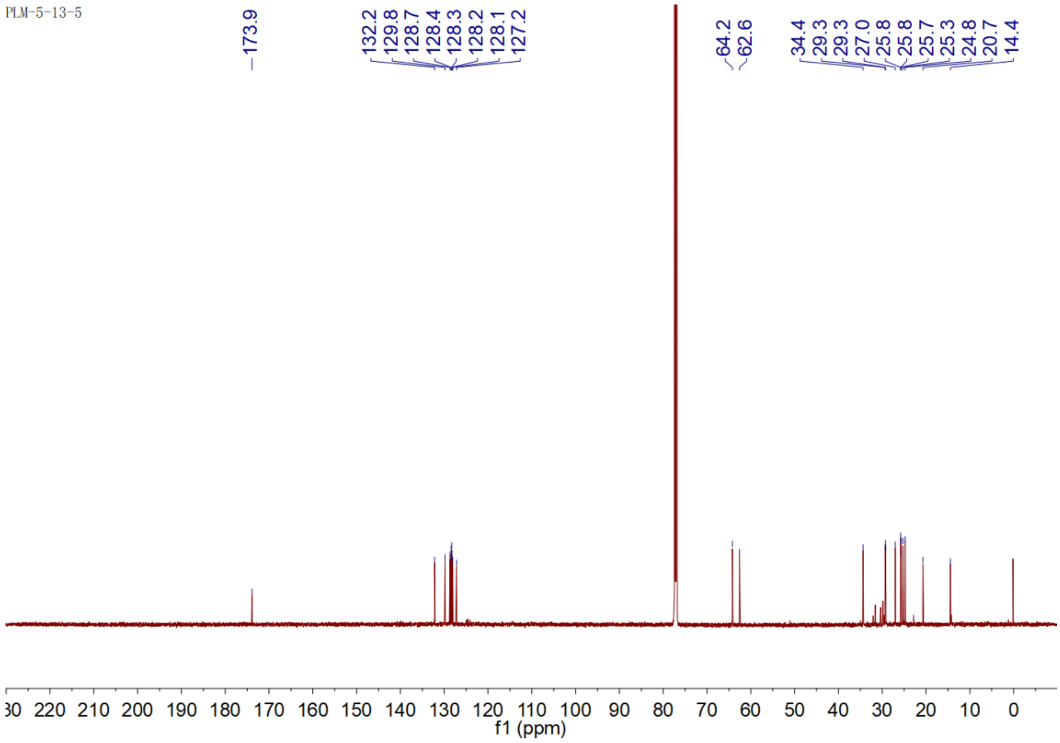

Figure S24 ^{13}C-NMR spectrum of compound **3**.

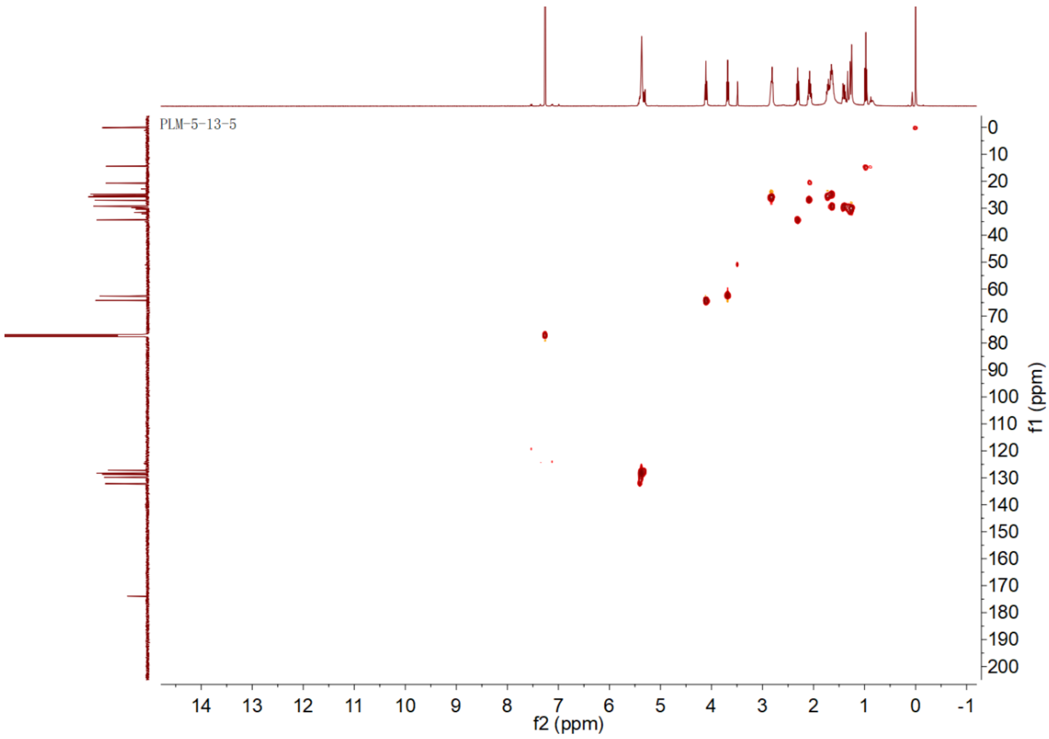

Figure S25 HSQC spectrum of compound **3**.

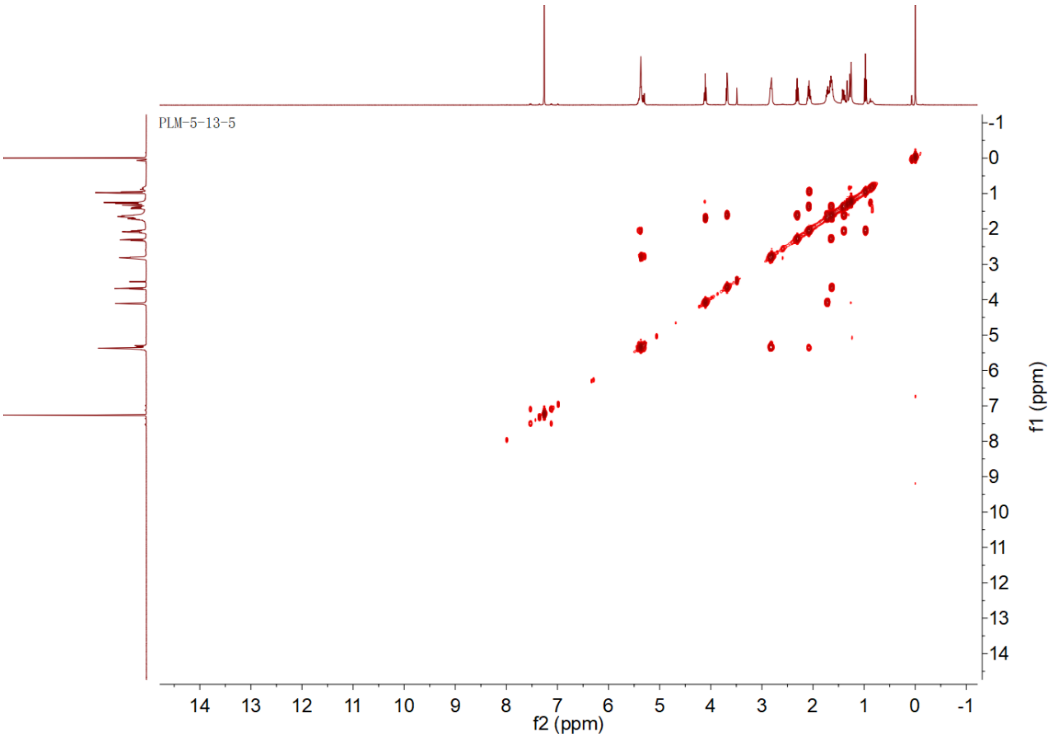

Figure S26 ^{1}H-^{1}H COSY spectrum of compound **3**.

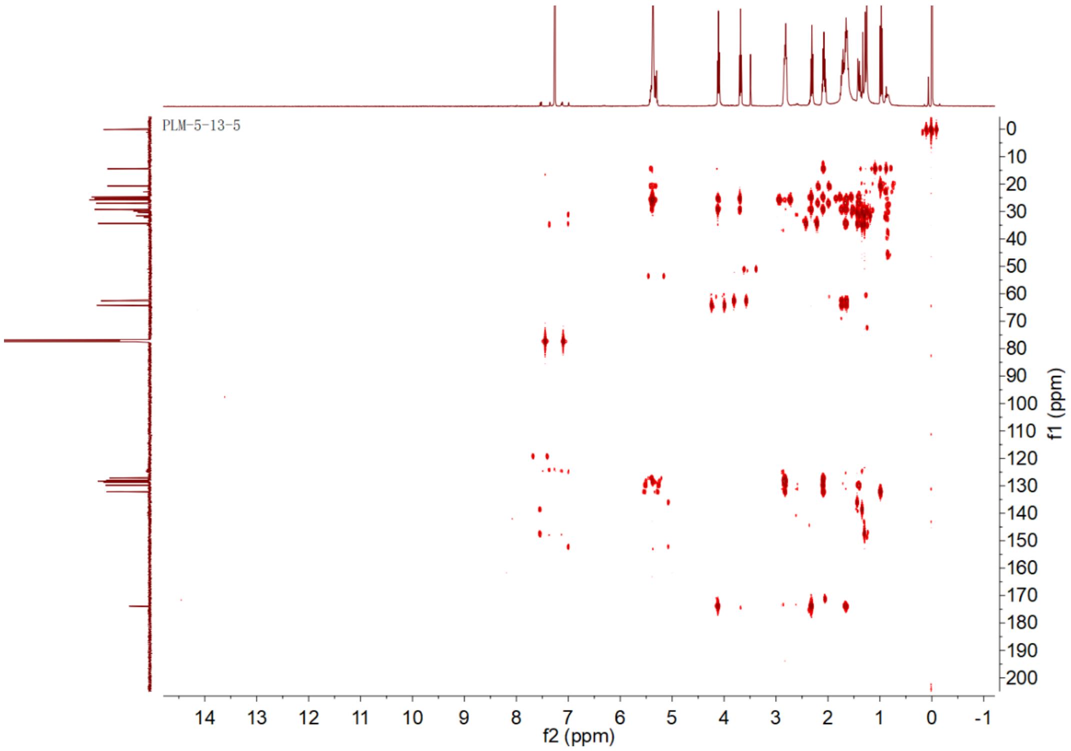

Figure S27 HMBC spectrum of compound **3**.

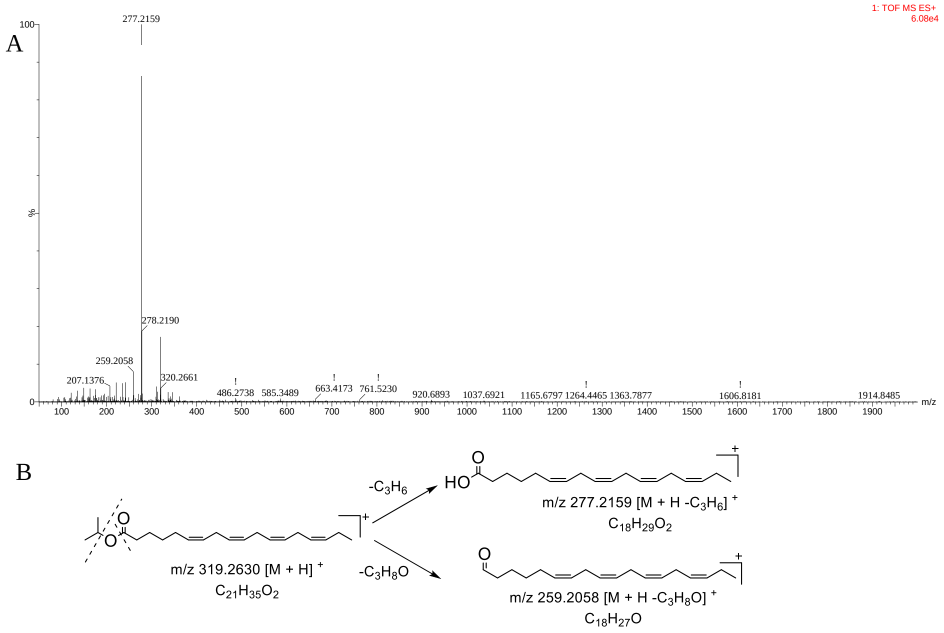

Figure S28 The MS spectrum (A) and fragmentation pathways (B) of compound **4**.

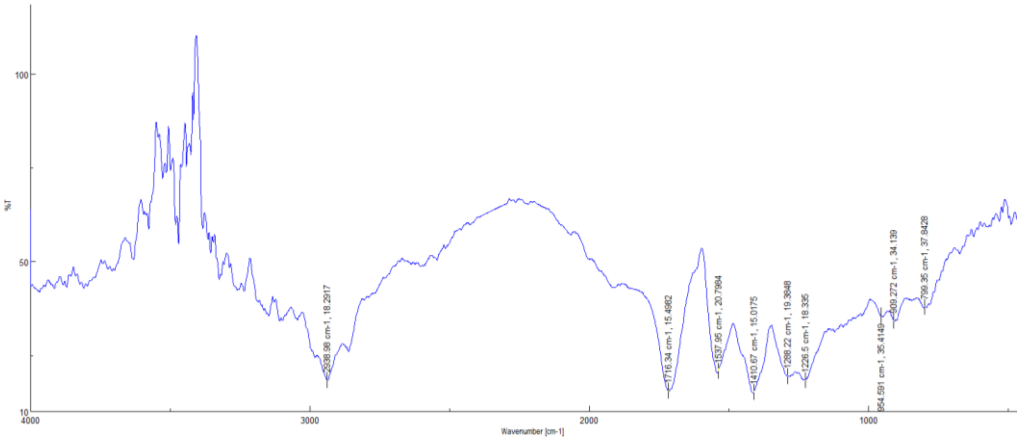

Figure S29 IR spectrum of compound **4**.

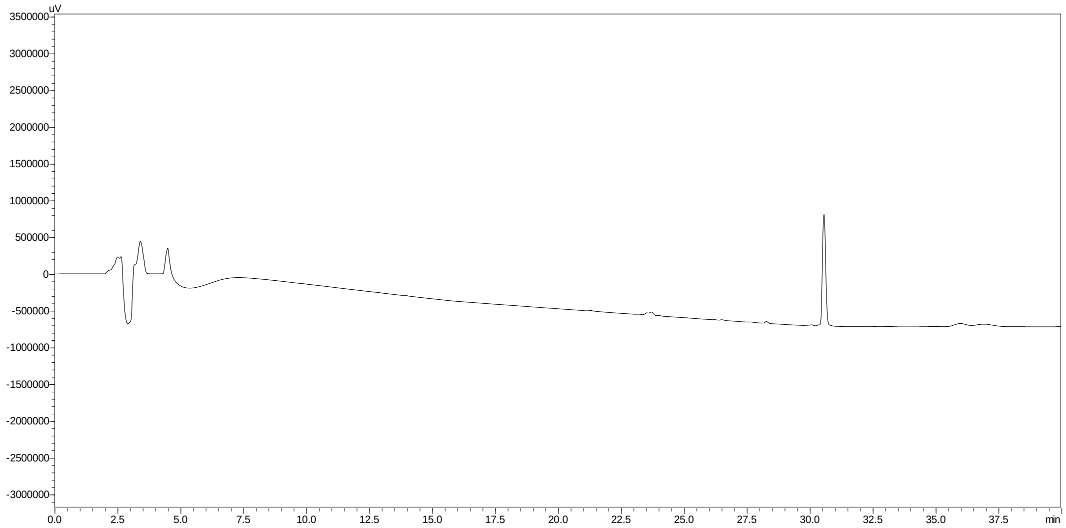

Figure S30 The HPLC results of compounds **4**.

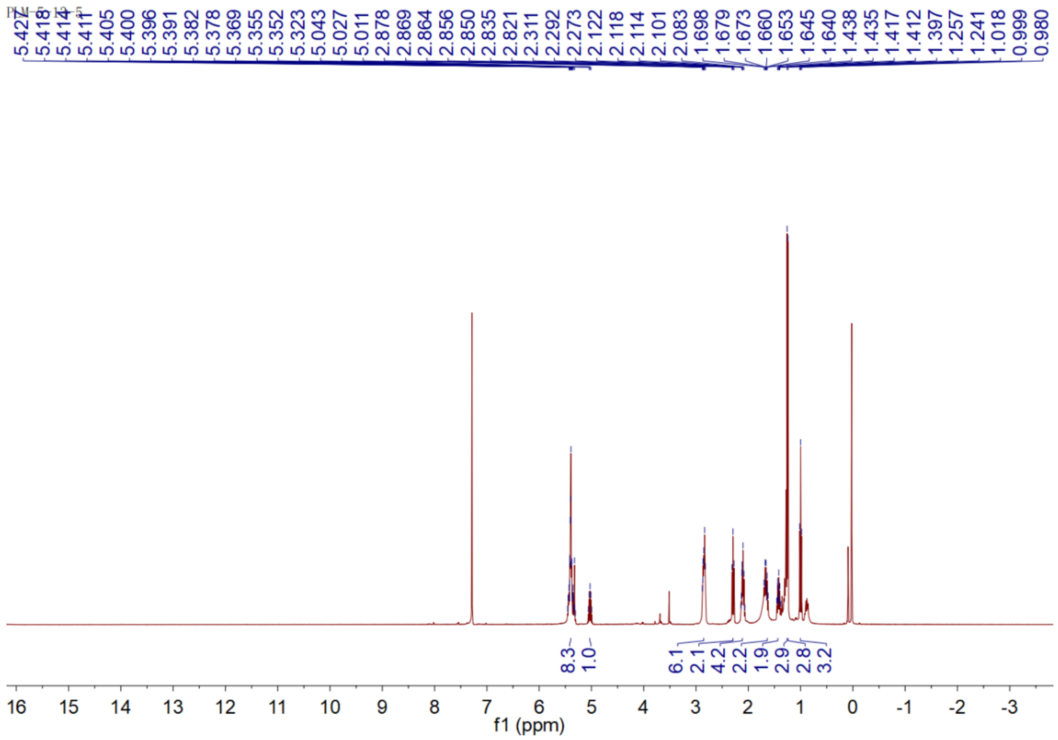

Figure S31 ^{1}H-NMR spectrum of compound **4**.

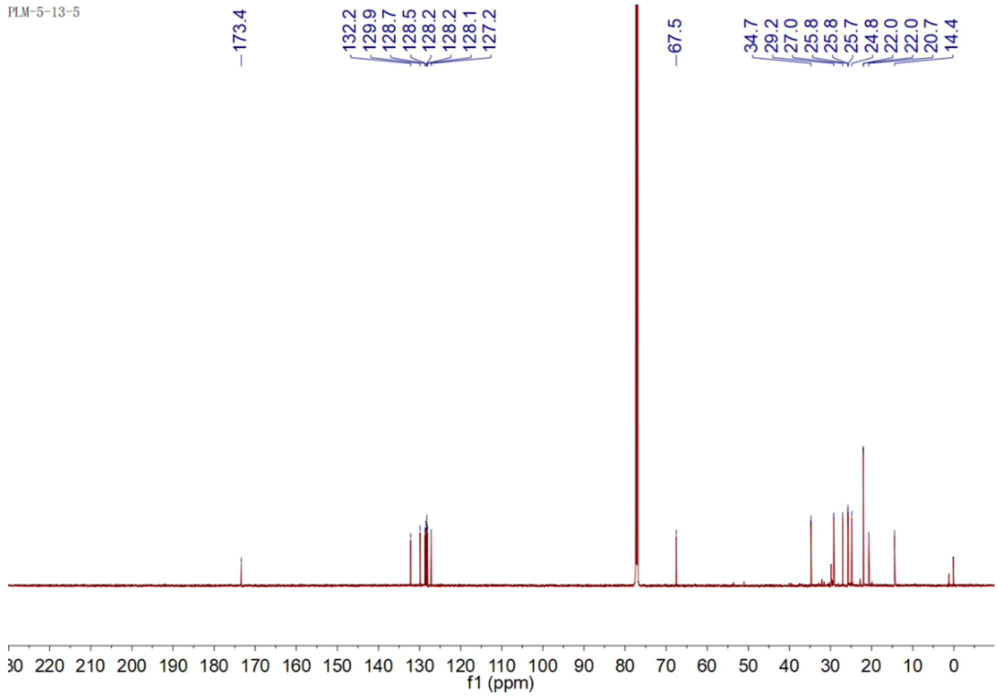

Figure S32 ^{13}C-NMR spectrum of compound **4**.

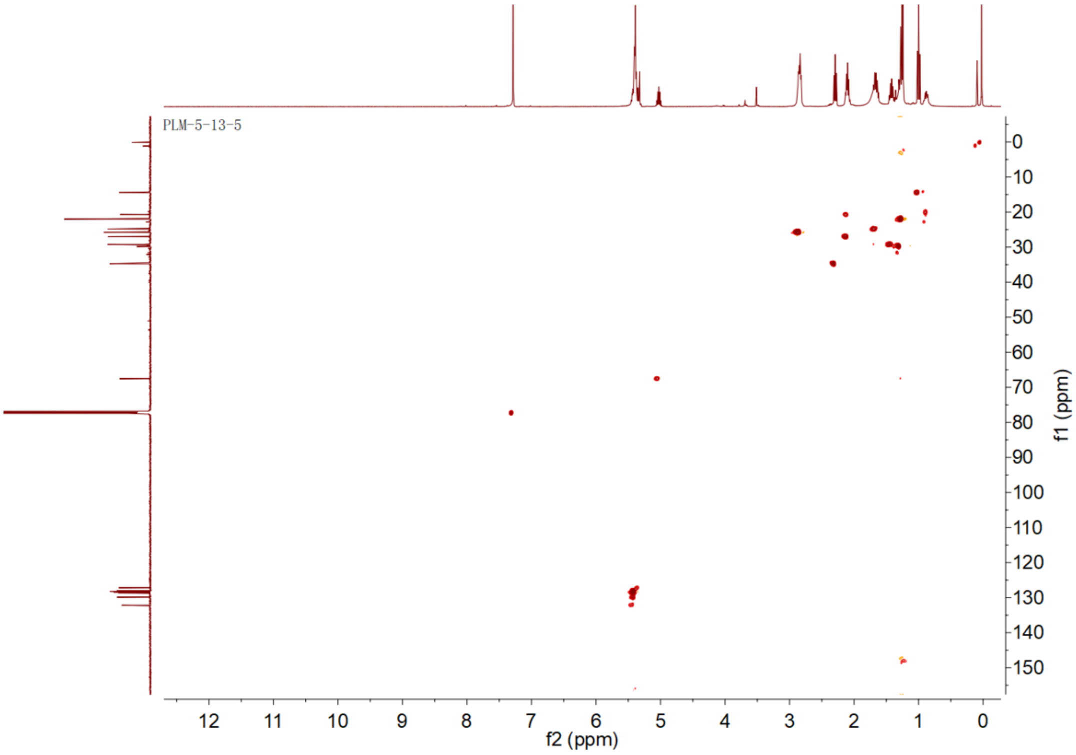

Figure S33 HSQC spectrum of compound **4**.

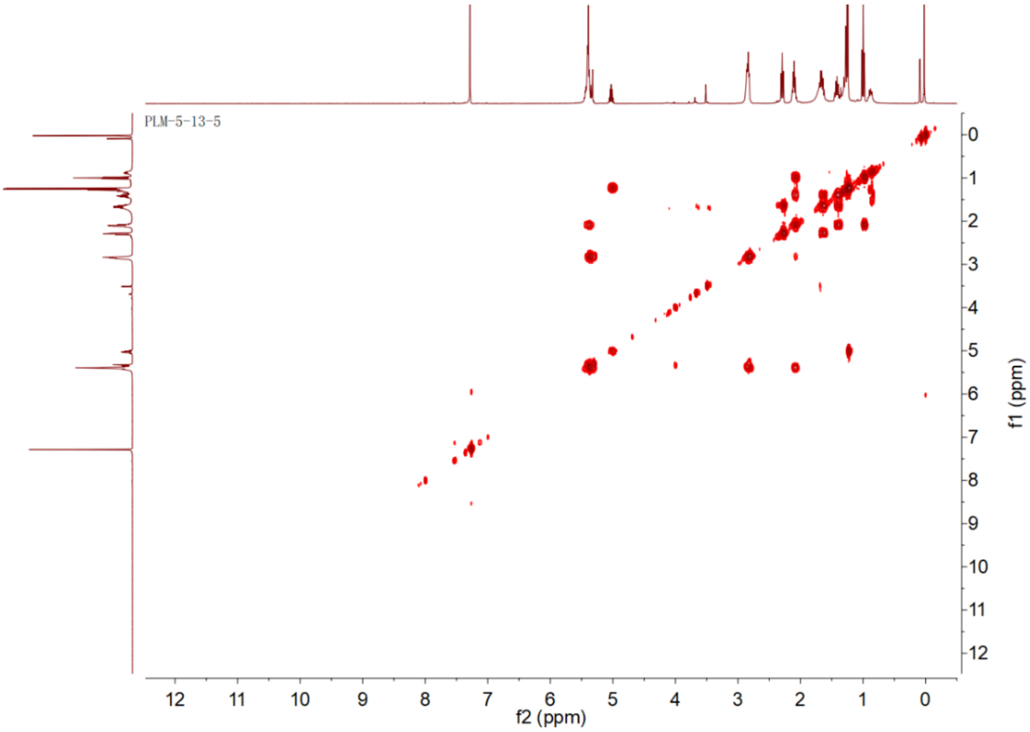

Figure S34 ^{1}H-^{1}H COSY spectrum of compound **4**.

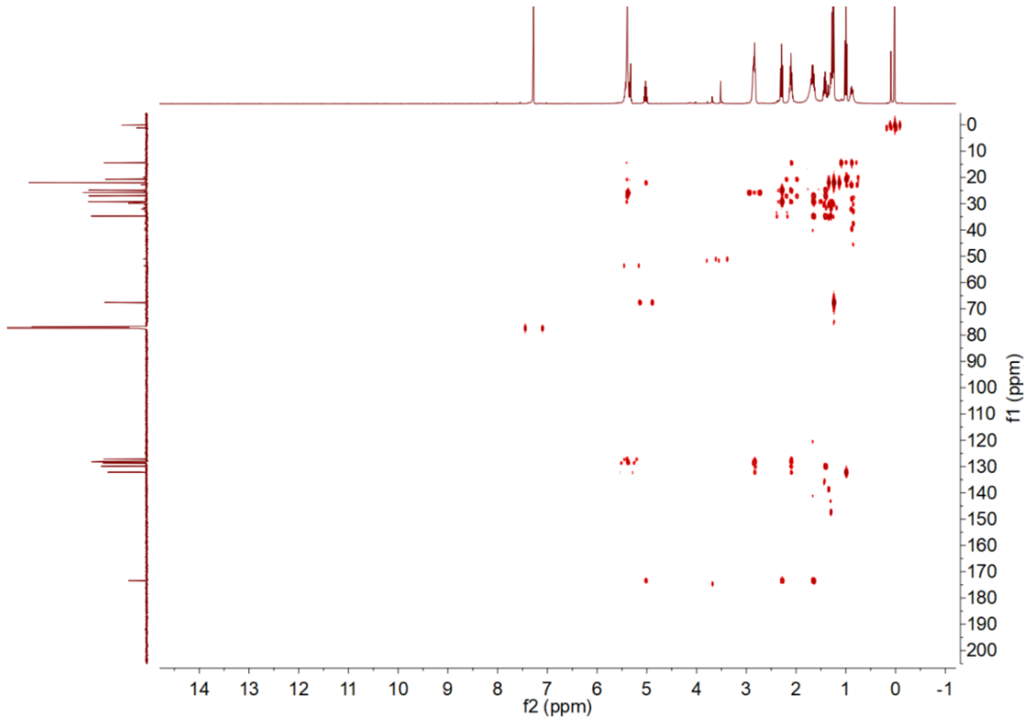

Figure S35 HMBC spectrum of compound **4**.

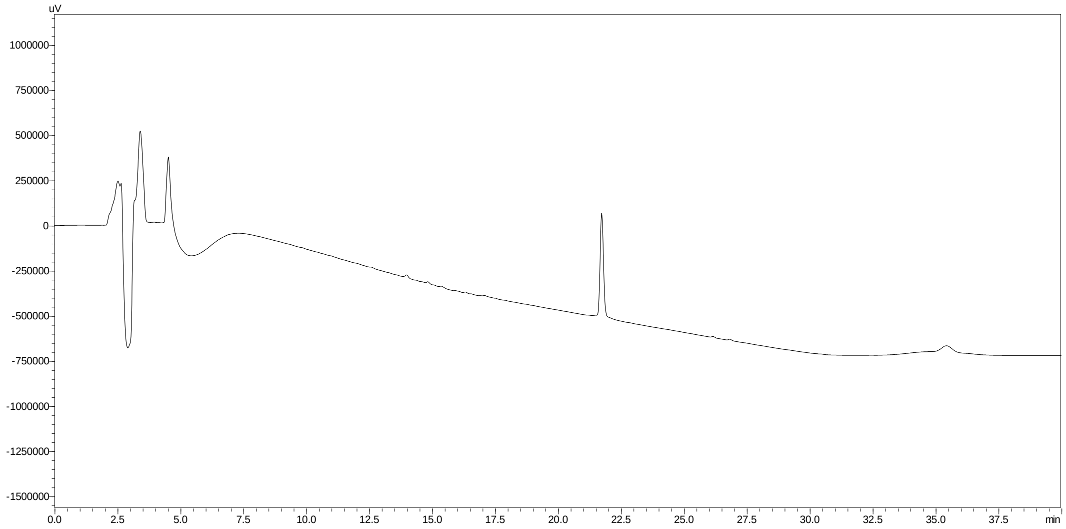

Figure S36 The HPLC results of compounds **5**.

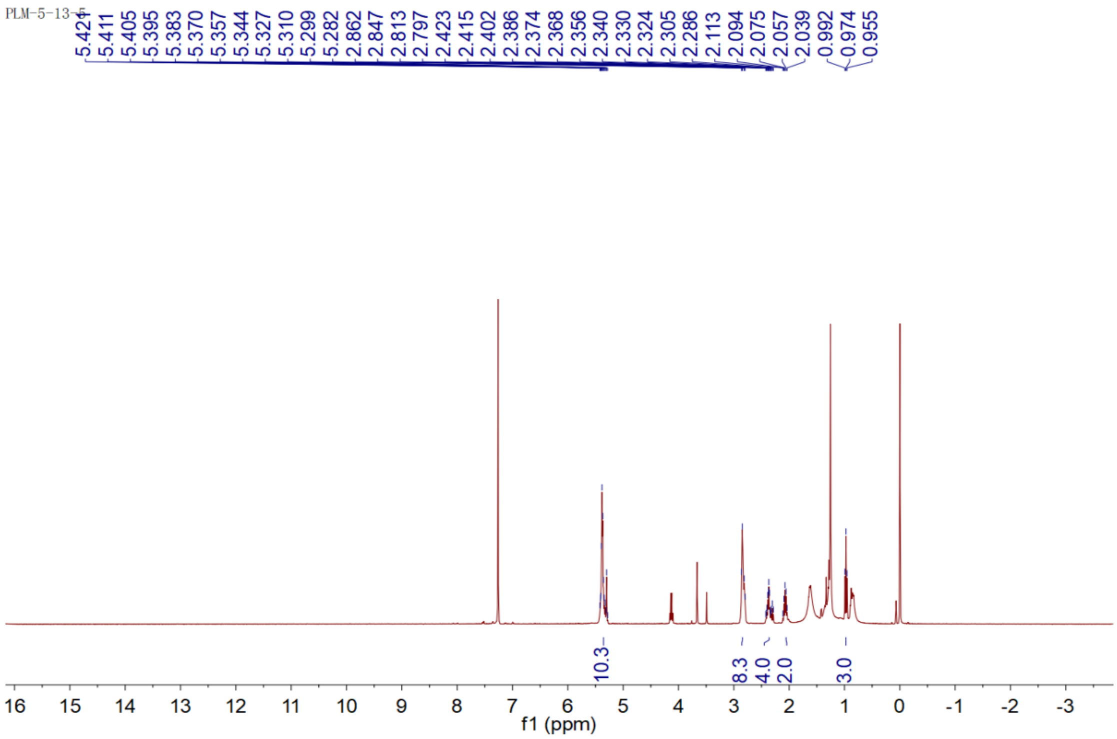

Figure S37 ^{1}H-NMR spectrum of compound **5**.

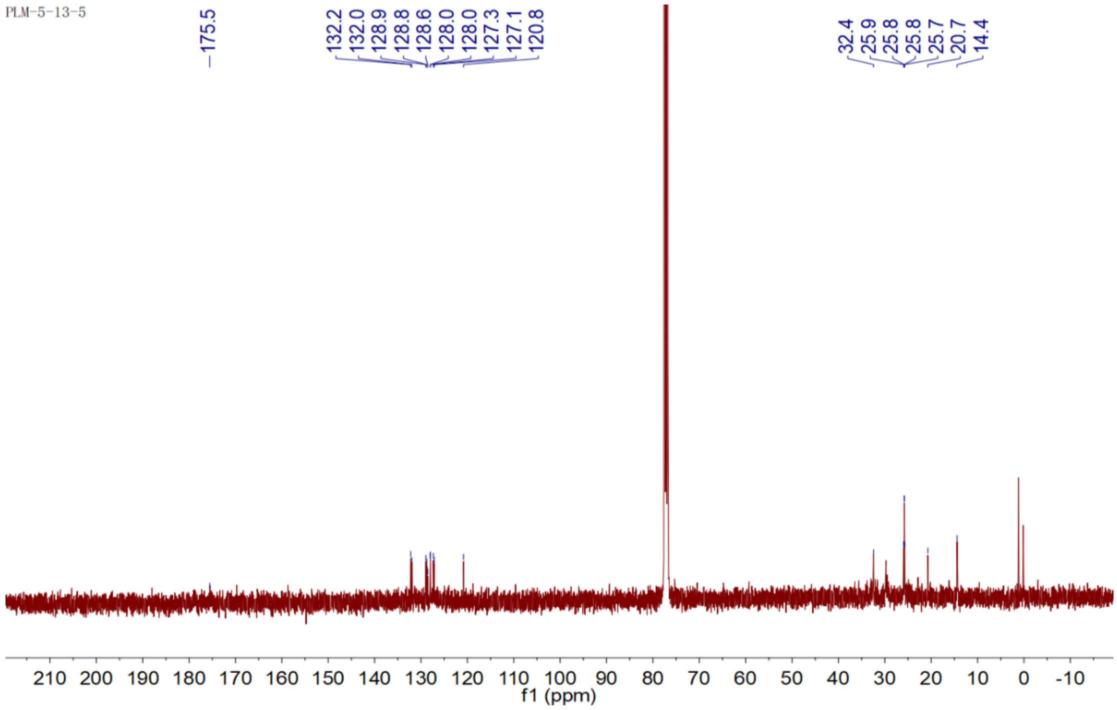

Figure S38 ^{13}C-NMR spectrum of compound **5**.

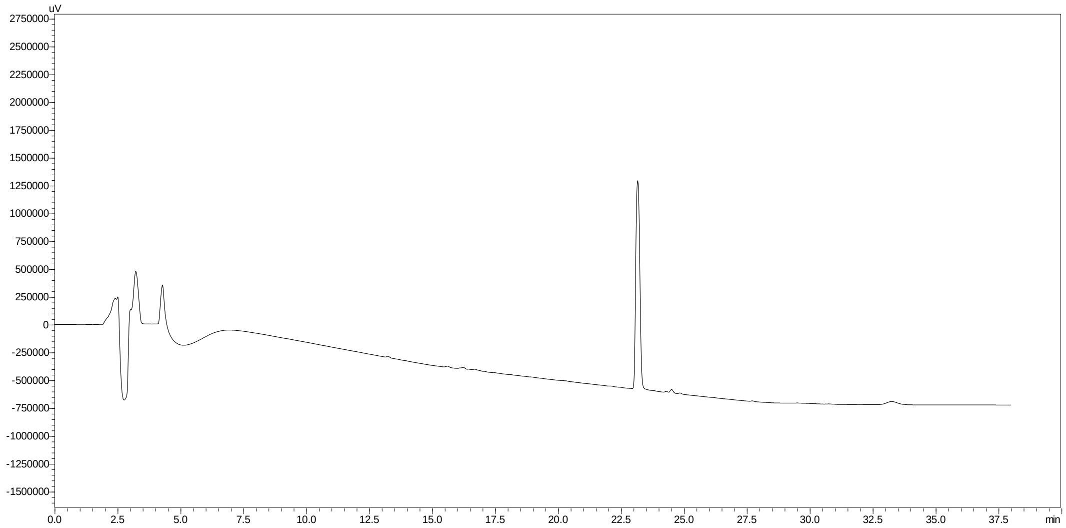

Figure S39 The HPLC results of compounds **6**.

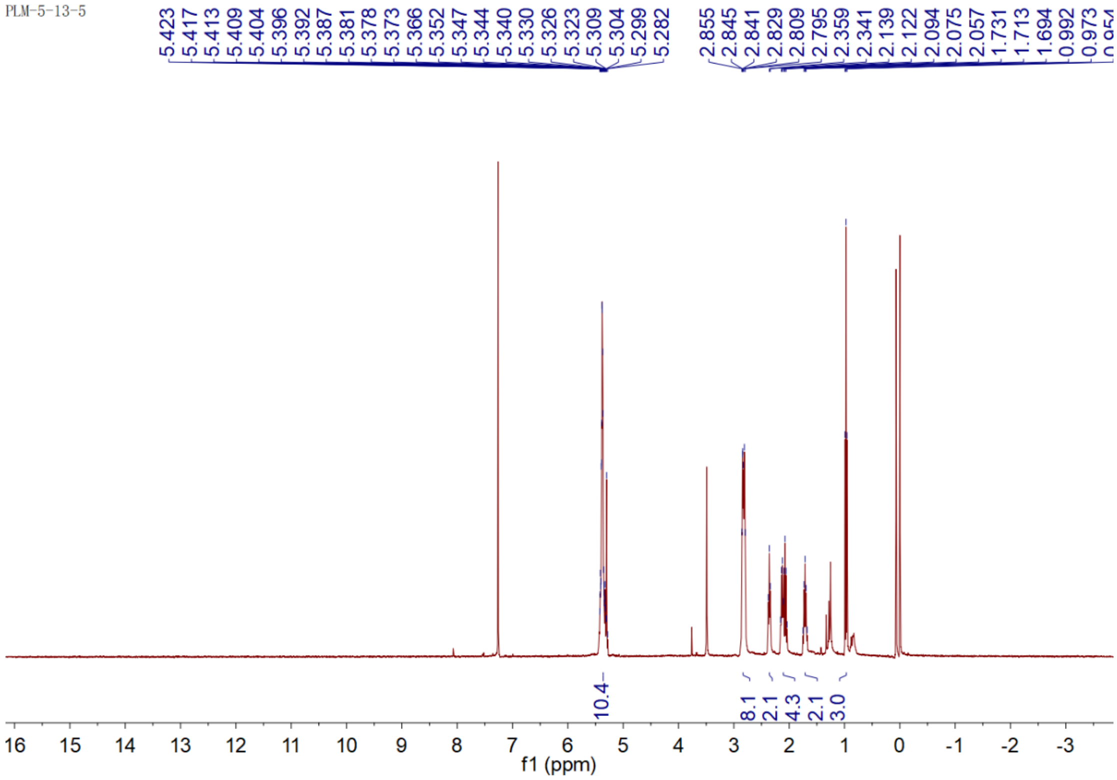

Figure S40 ^{1}H-NMR spectrum of compound **6**.

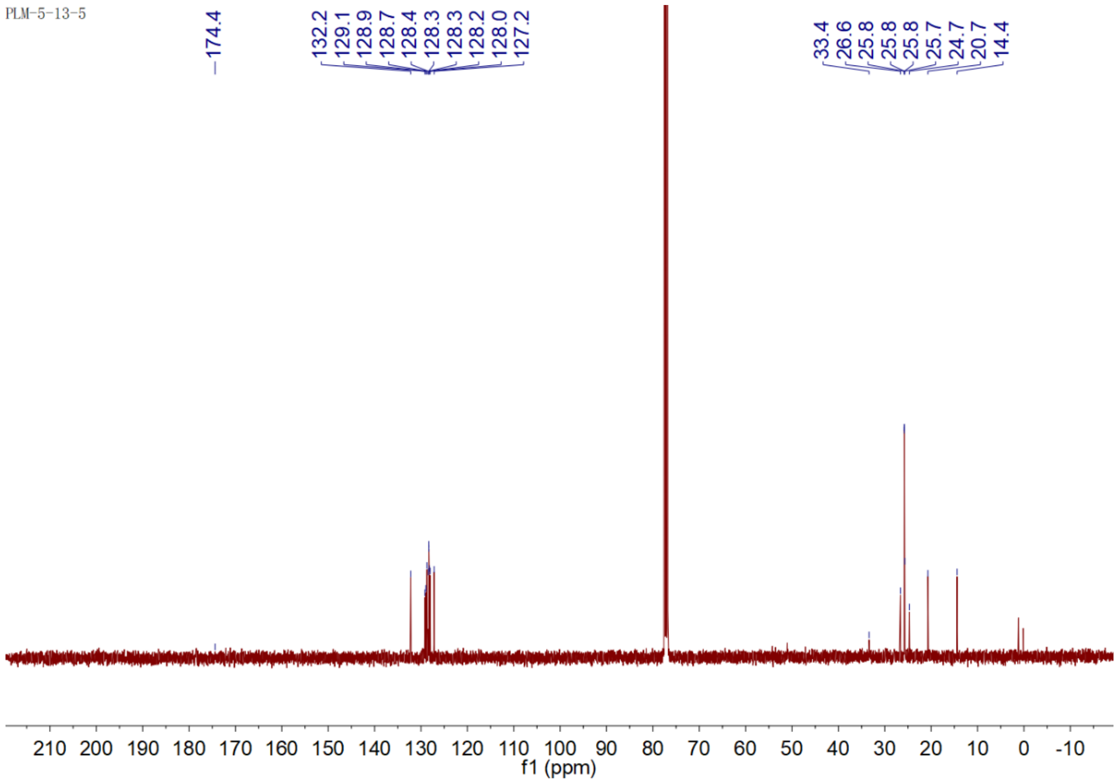

Figure S41 ^{13}C-NMR spectrum of compound **6**.

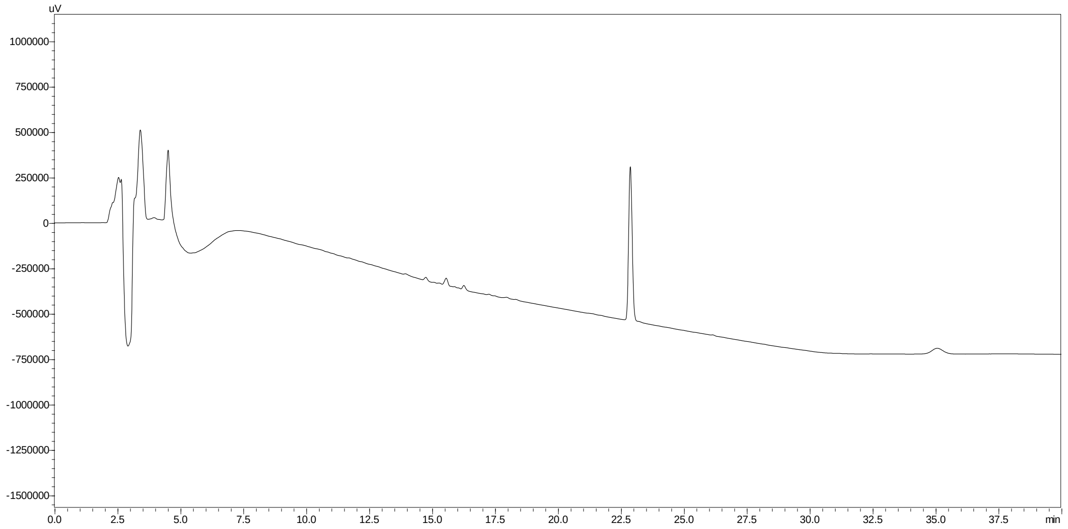

Figure S42 The HPLC results of compounds **7**.

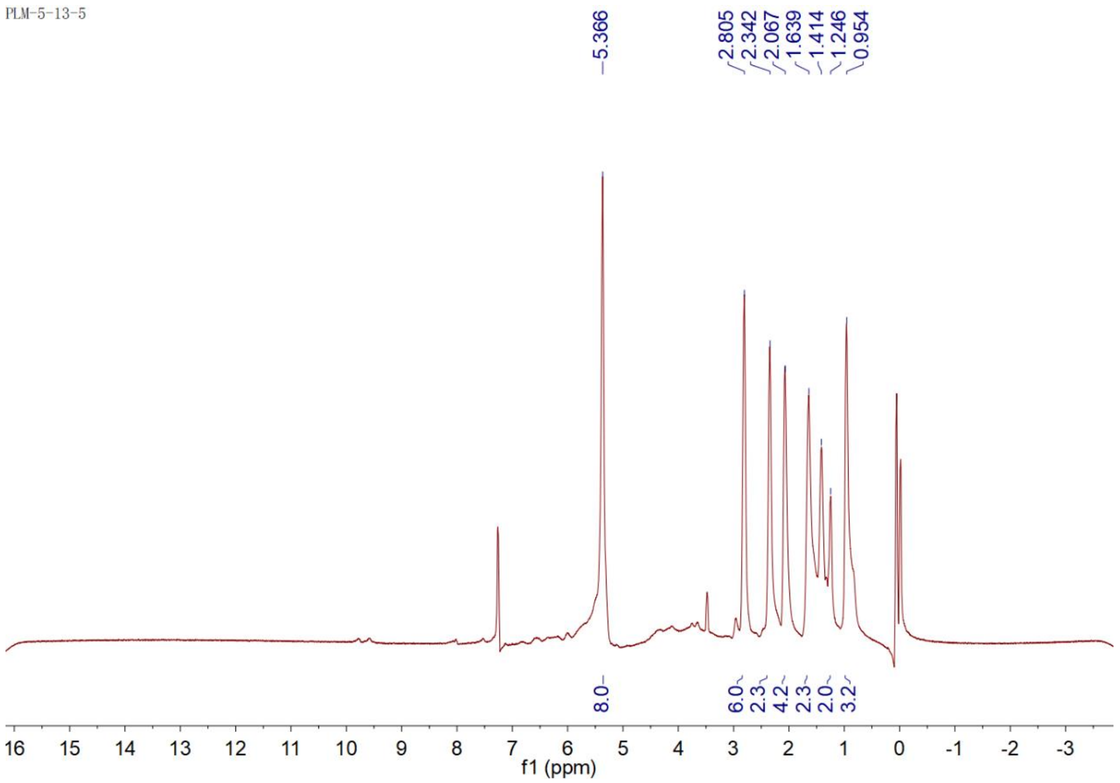

Figure S43 ^{1}H-NMR spectrum of compound **7**.

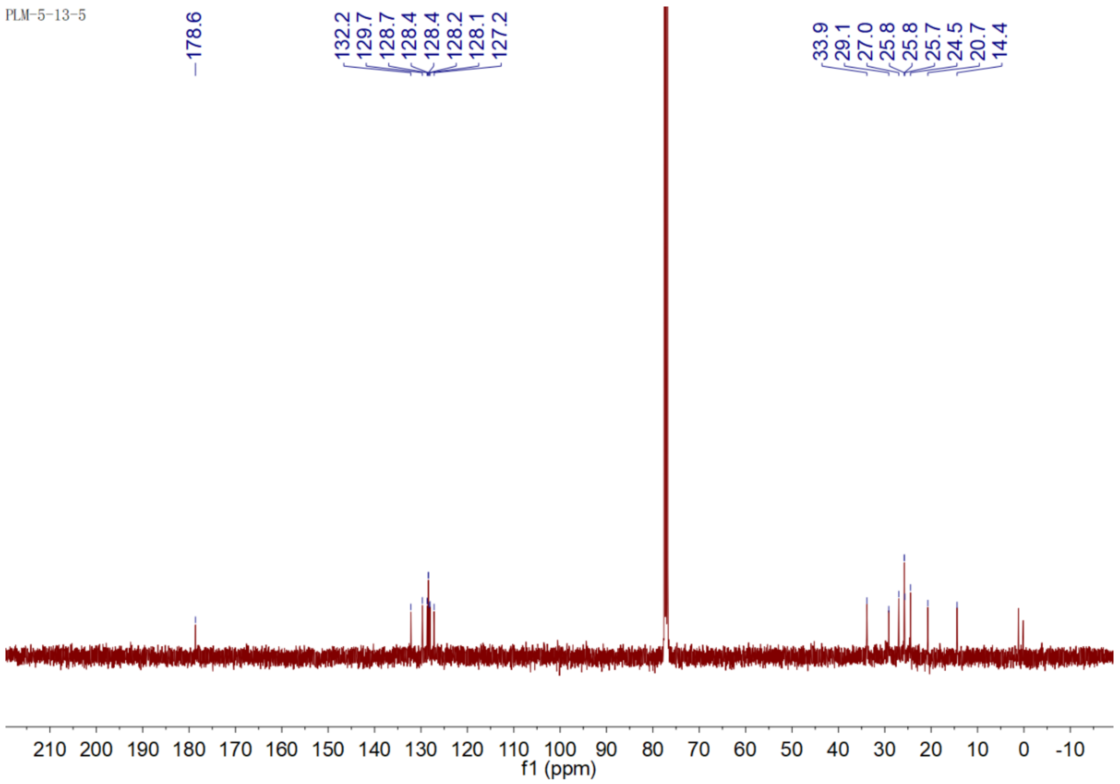

Figure S44 ^{13}C-NMR spectrum of compound **7**.

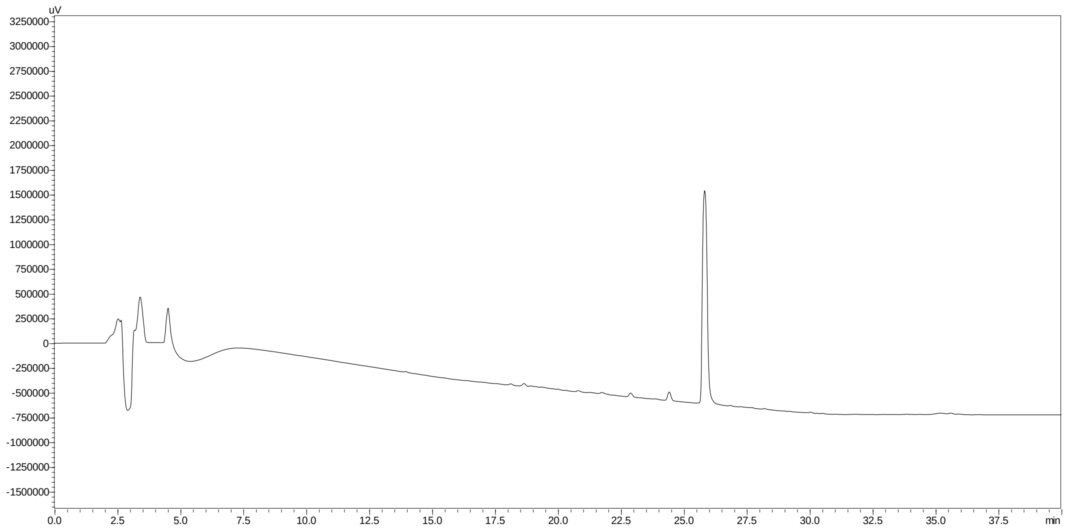

Figure S45 The HPLC results of compounds **8**.

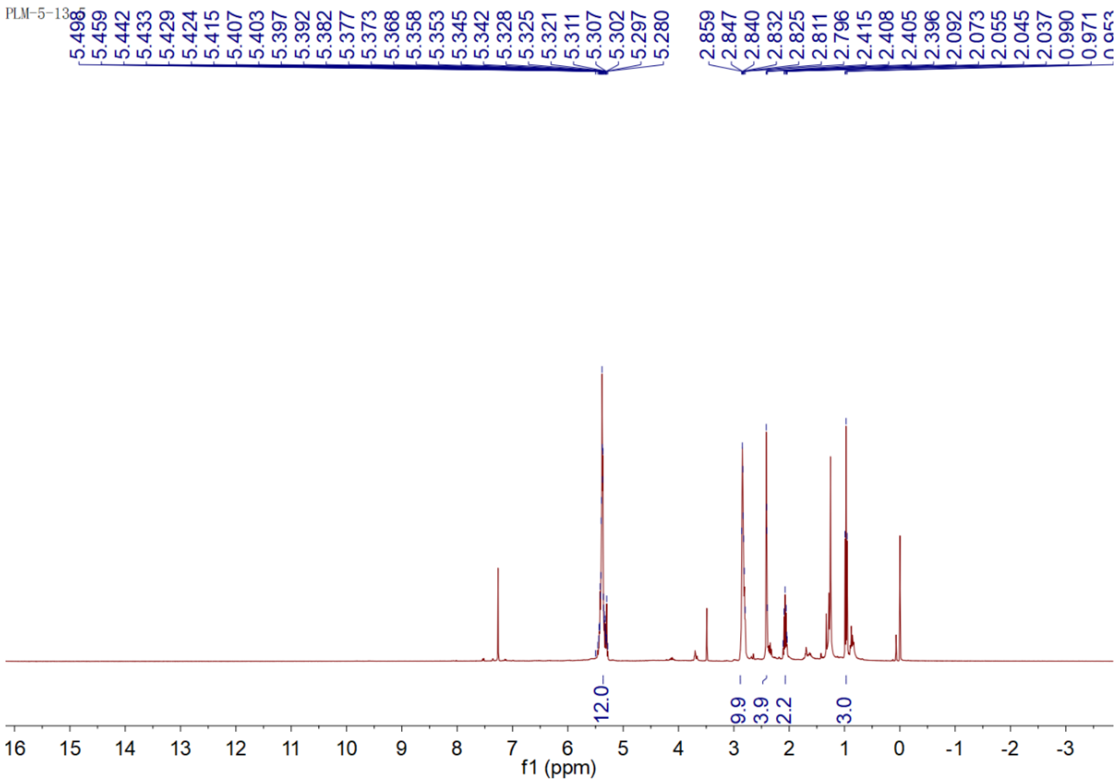

Figure S46 ^{1}H-NMR spectrum of compound **8**.

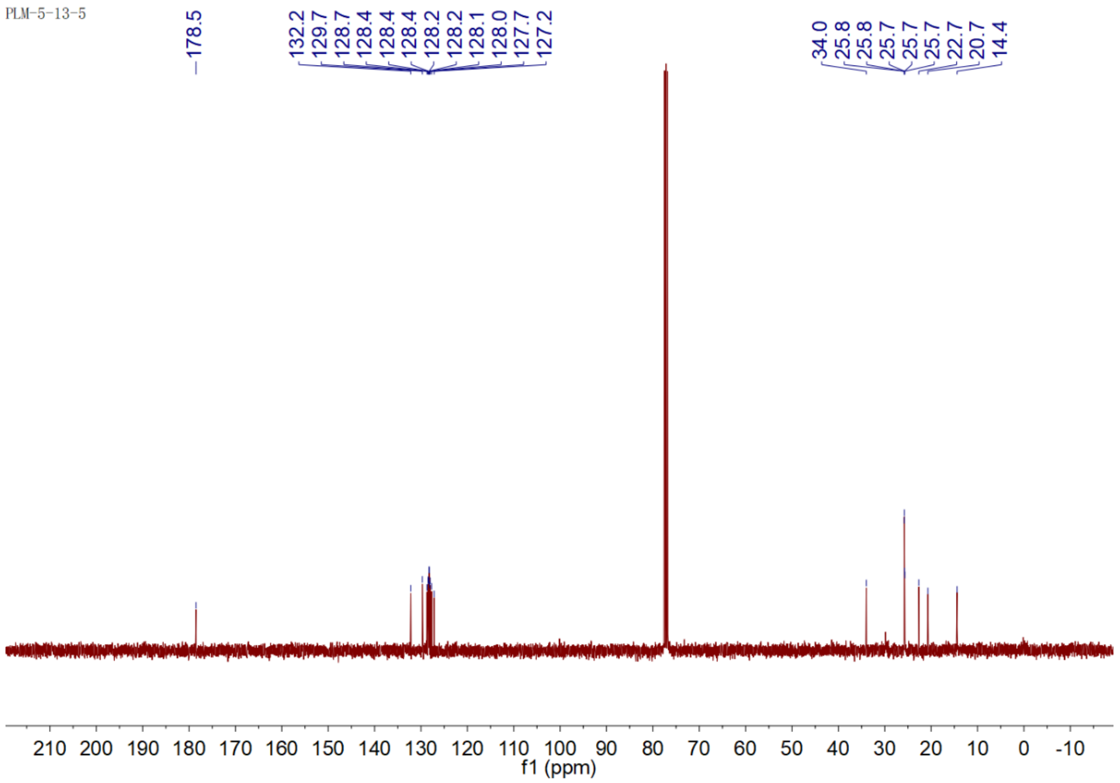

Figure S47 ^{13}C-NMR spectrum of compound **8**.

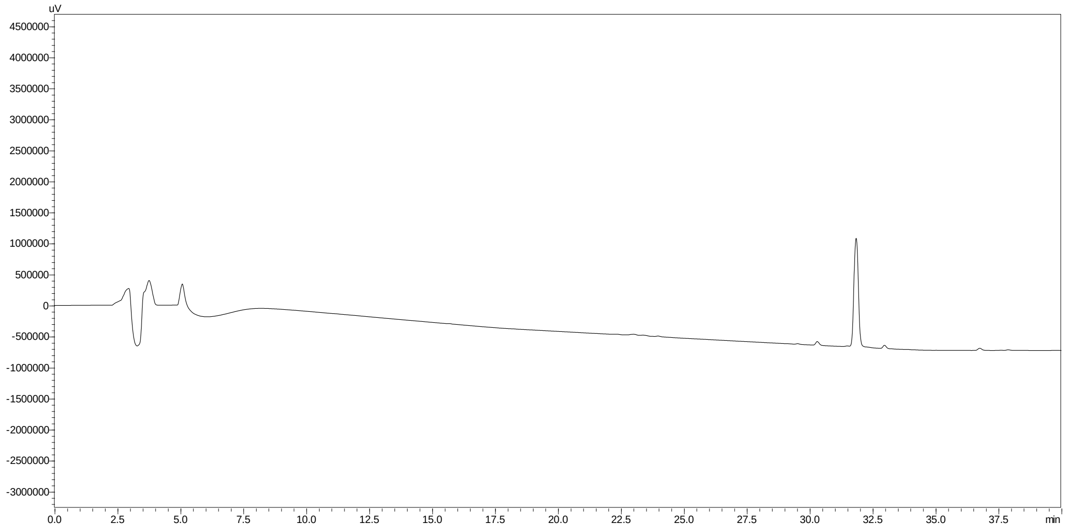

Figure S48 The HPLC results of compounds **9**.

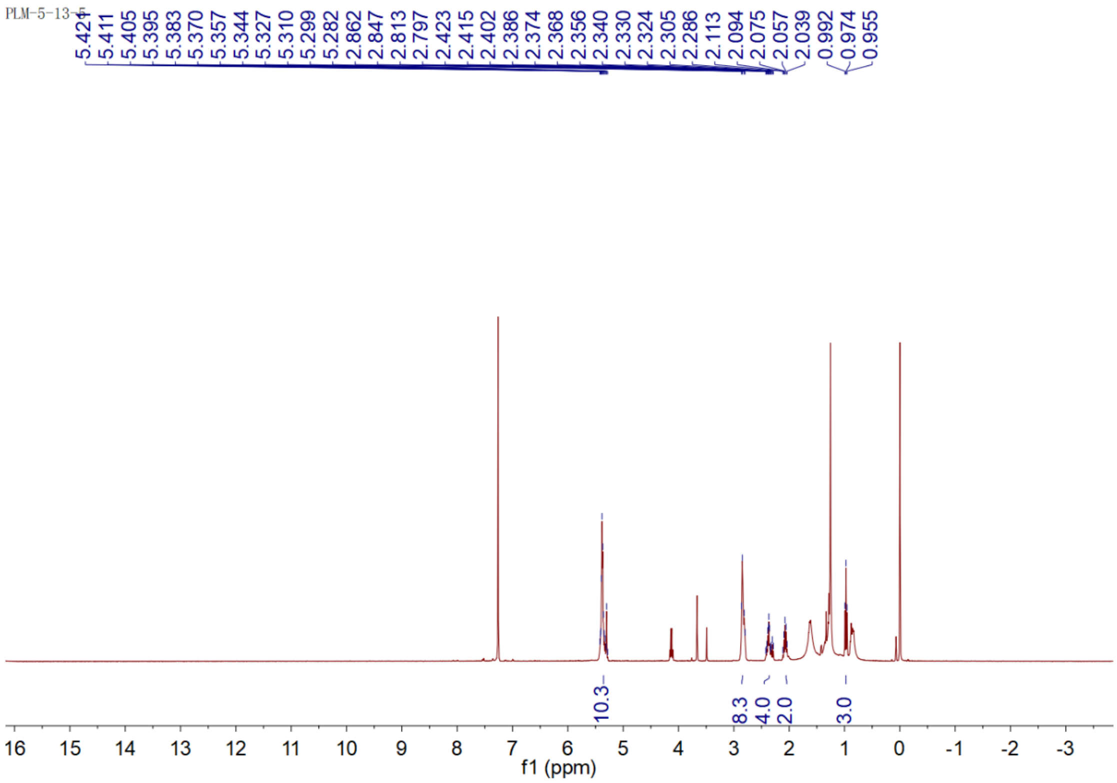

Figure S49 ^{1}H-NMR spectrum of compound **9**.

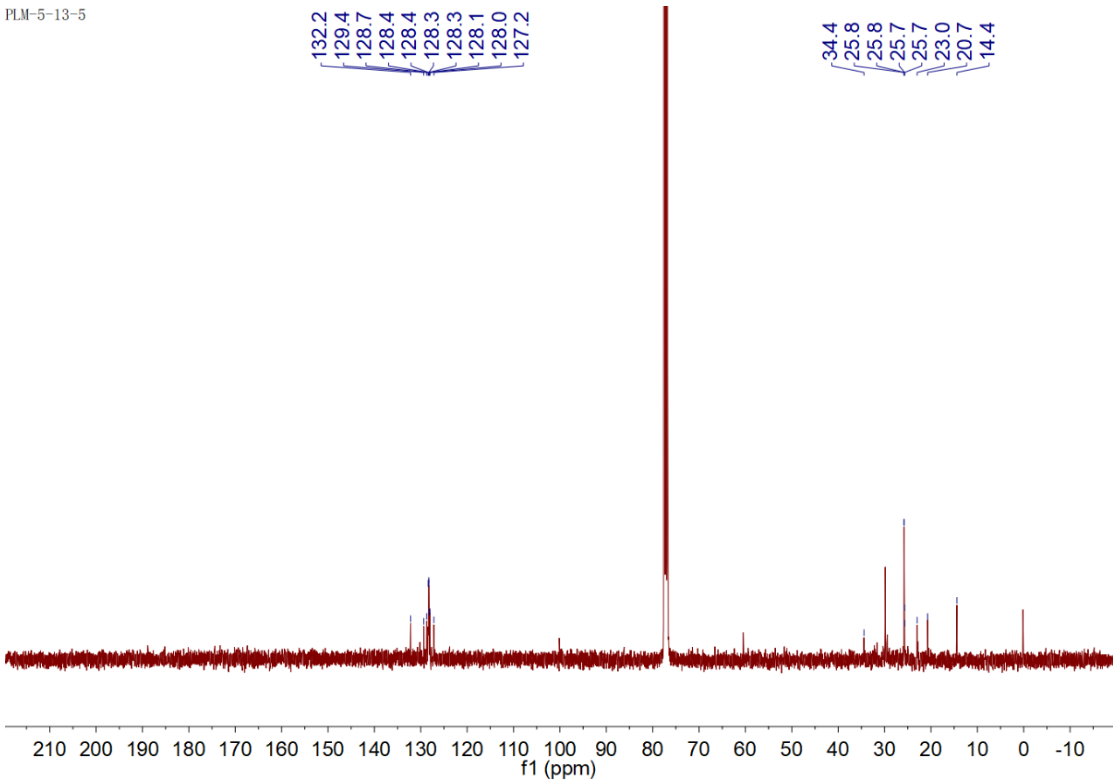

Figure S50 ^{13}C-NMR spectrum of compound **9**.

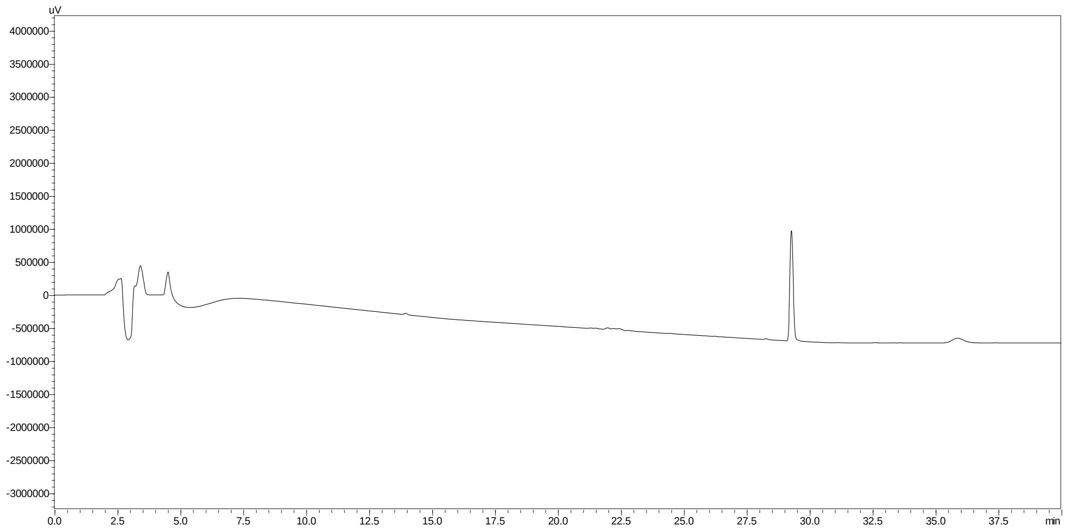

Figure S51 The HPLC results of compounds **10**.

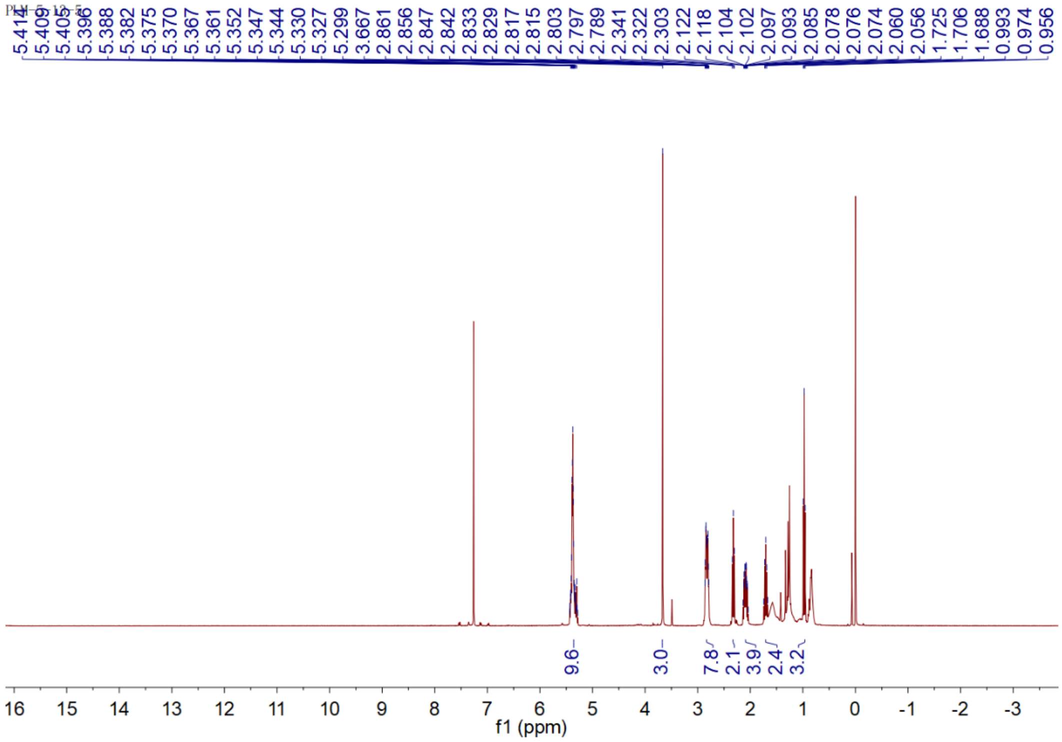

Figure S52 ^{1}H-NMR spectrum of compound **10**.

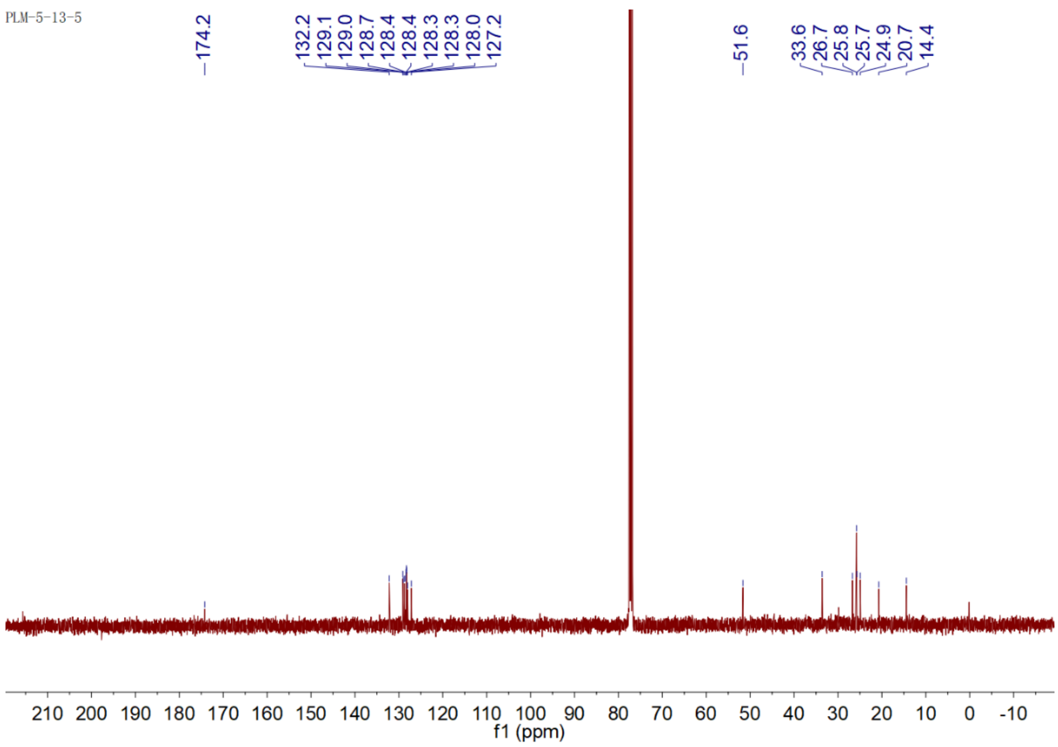

Figure S53 ^{13}C-NMR spectrum of compound **10**.

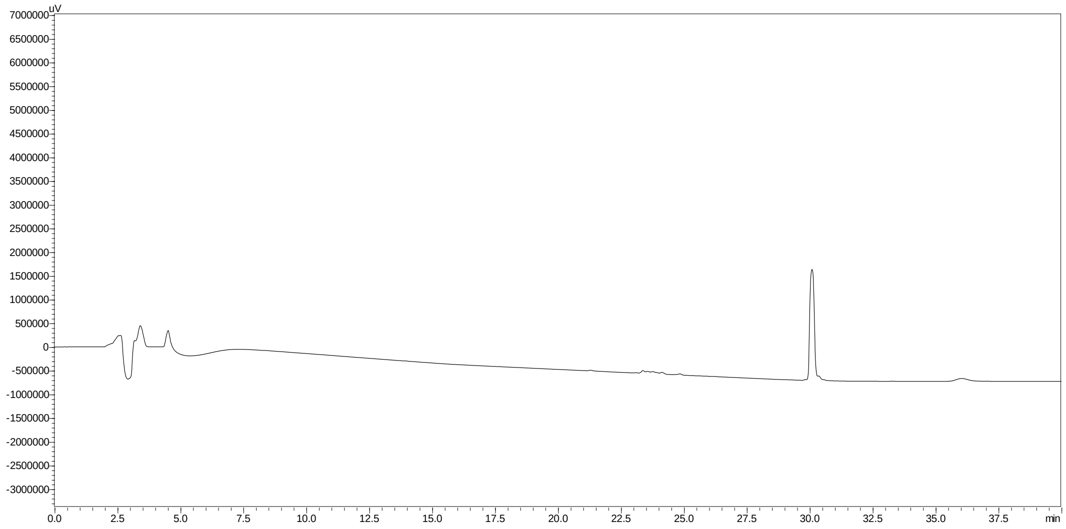

Figure S54 The HPLC results of compounds **11**.

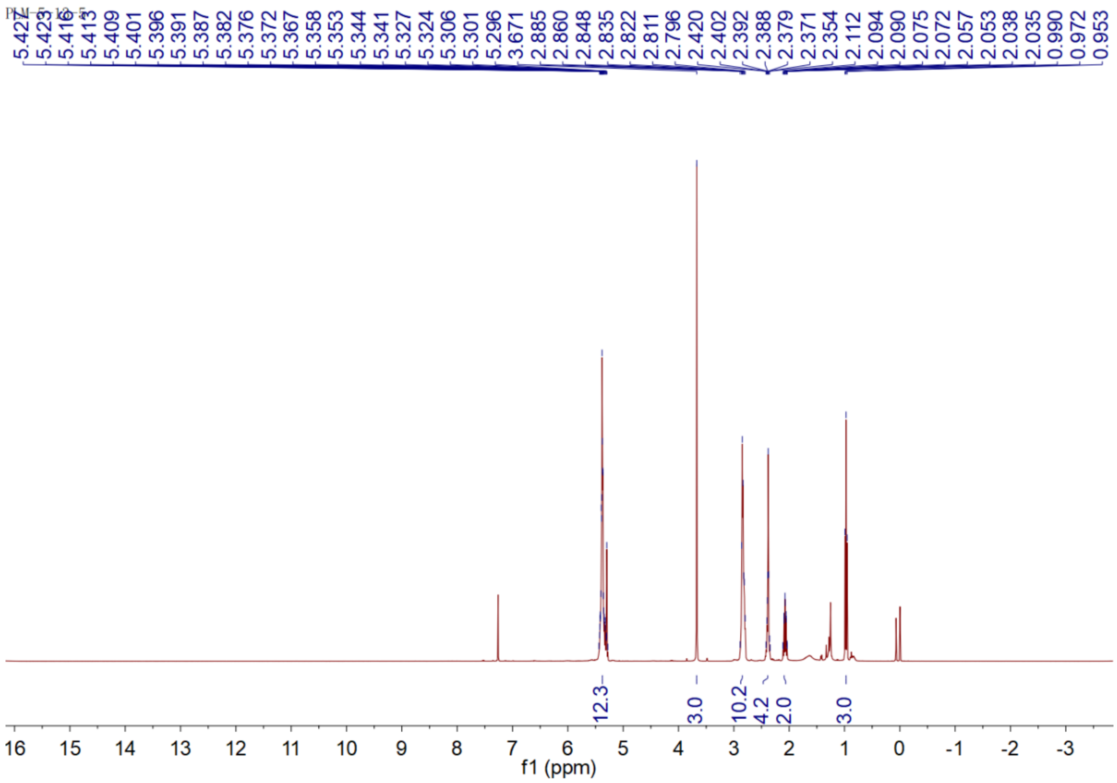

Figure S55 ^{1}H-NMR spectrum of compound **11**.

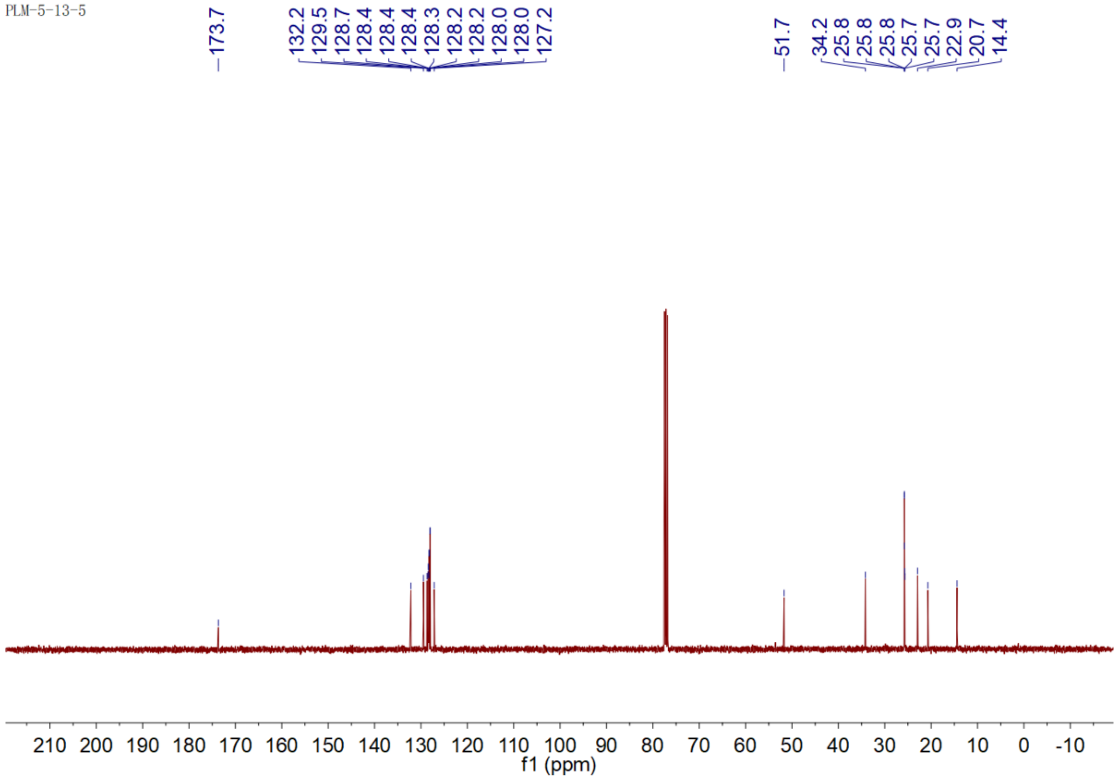

Figure S56 ^{13}C-NMR spectrum of compound **11**.

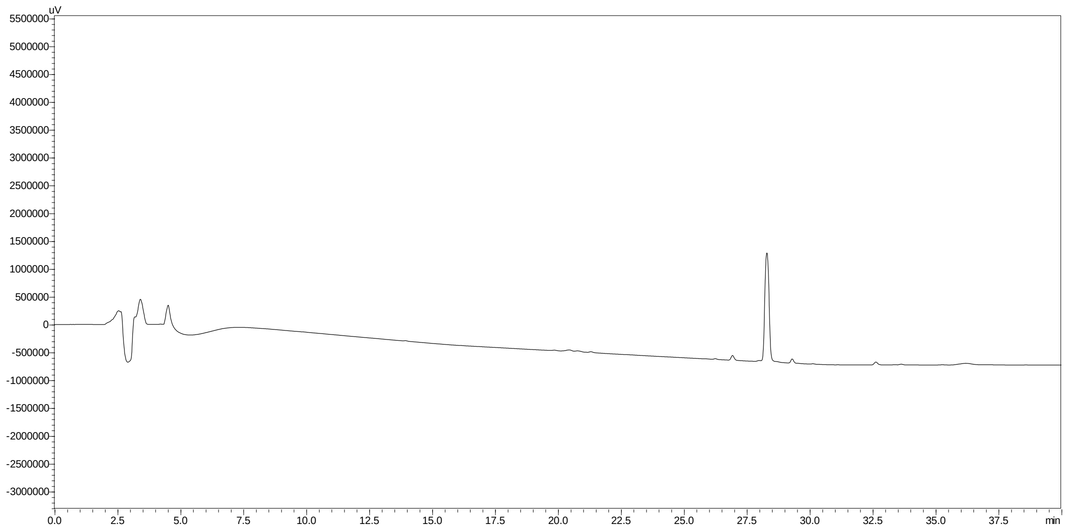

Figure S57 The HPLC results of compounds **12**.

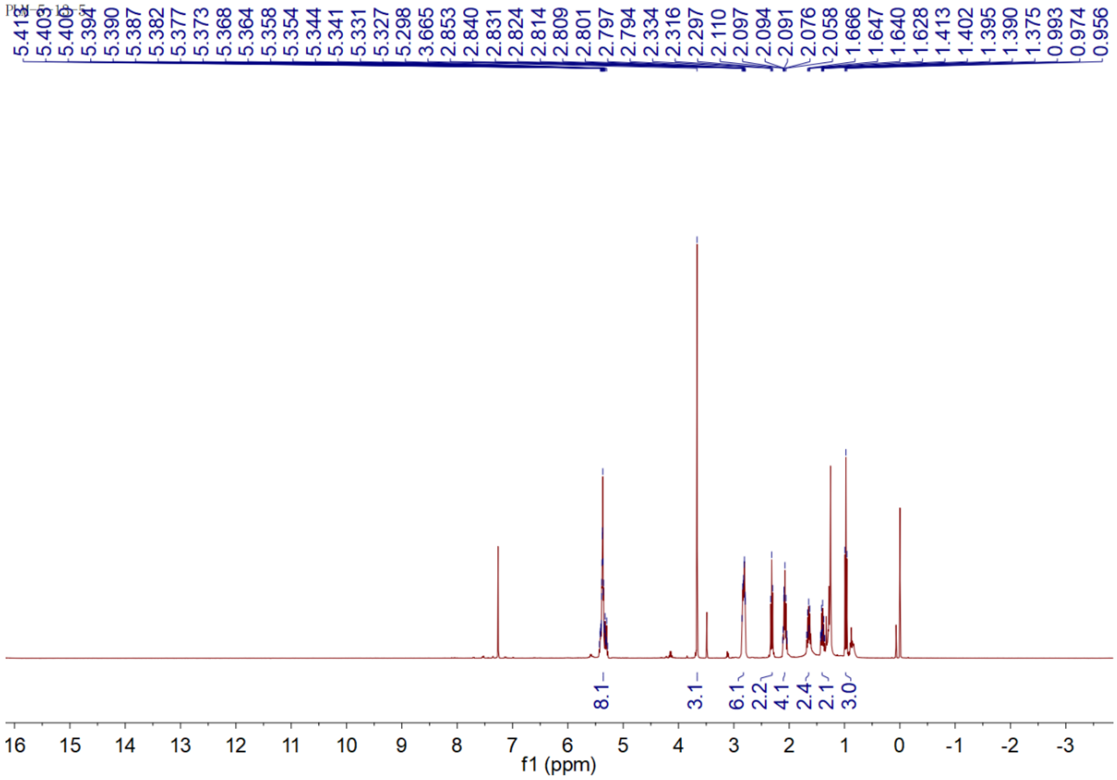

Figure S58 ^{1}H-NMR spectrum of compound **12**.

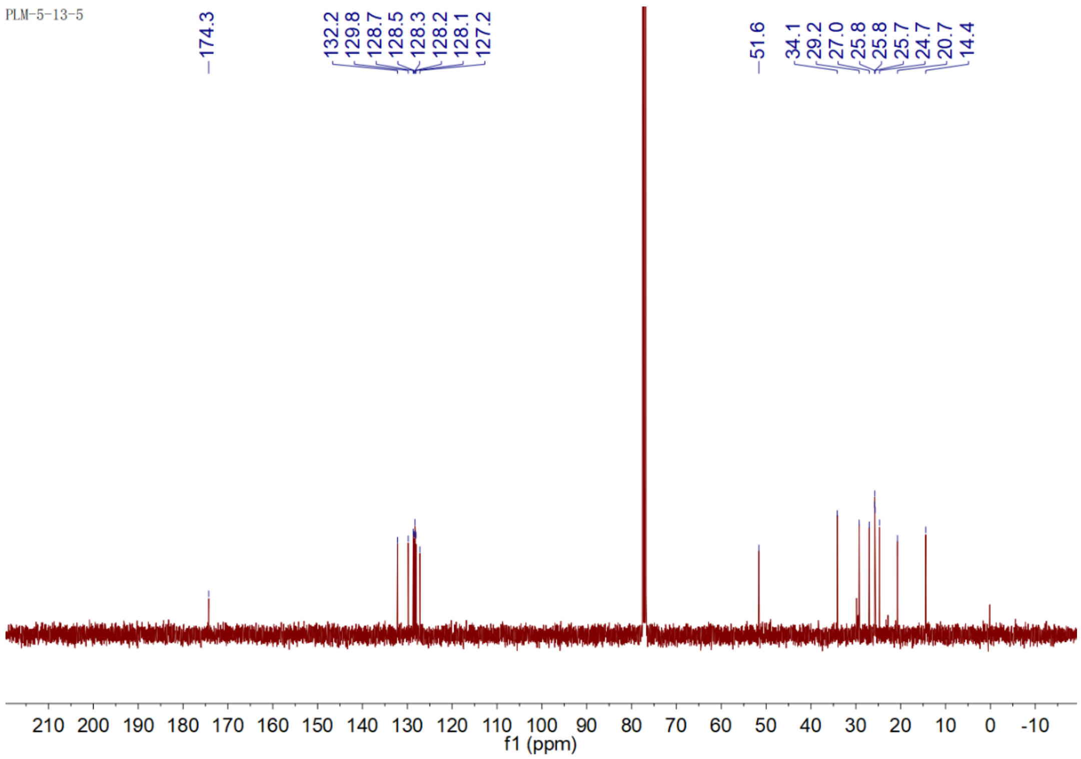

Figure S59 ^{13}C-NMR spectrum of compound **12**.

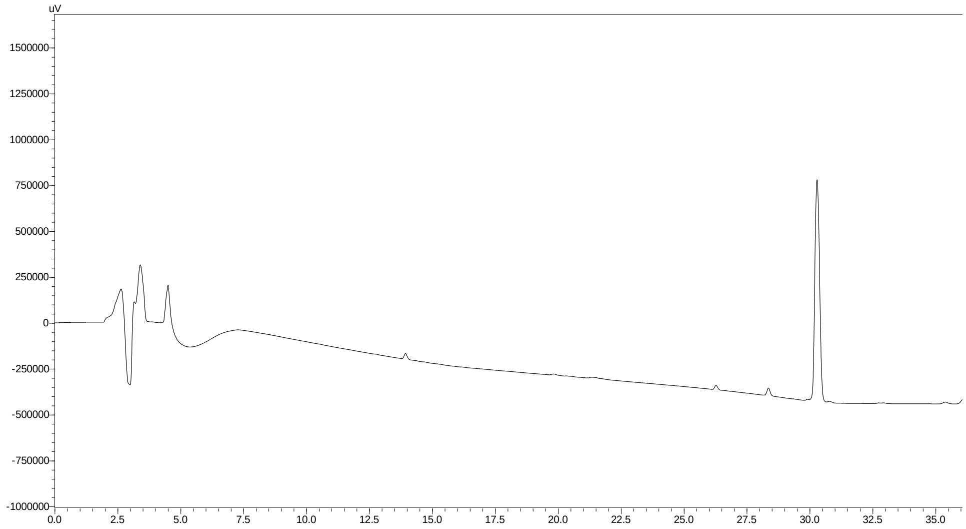

Figure S60 The HPLC results of compounds **13**.

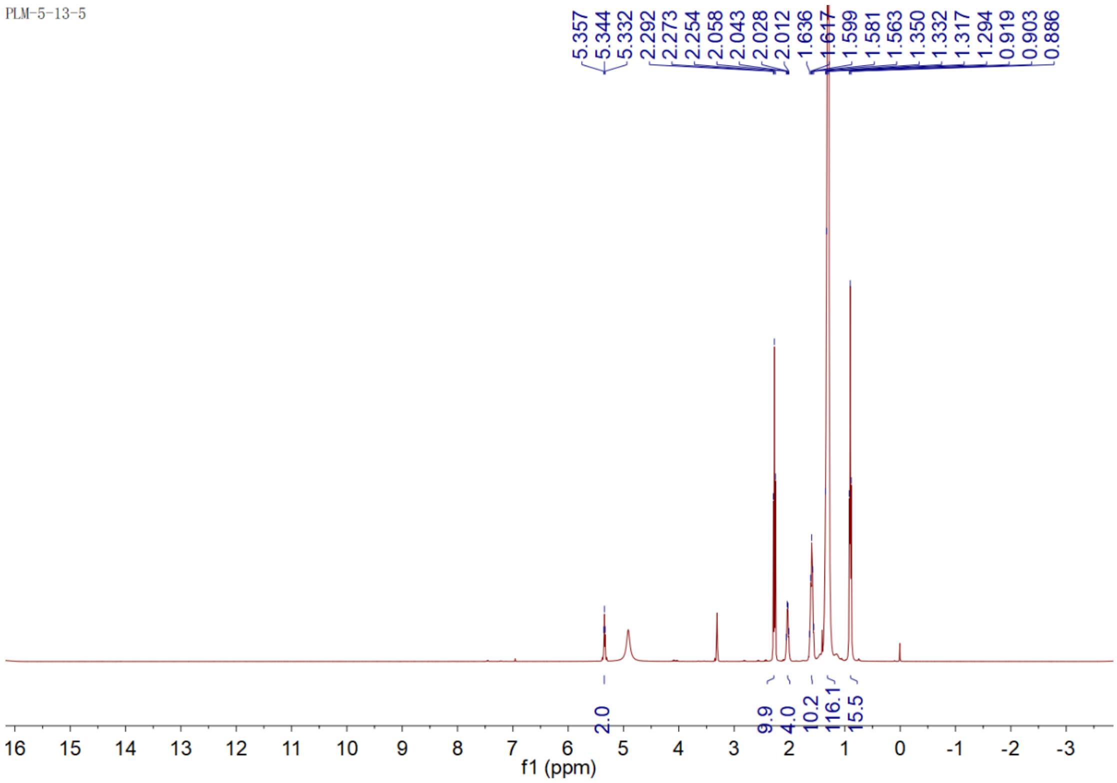

Figure S61 ^{1}H-NMR spectrum of compound **13**.

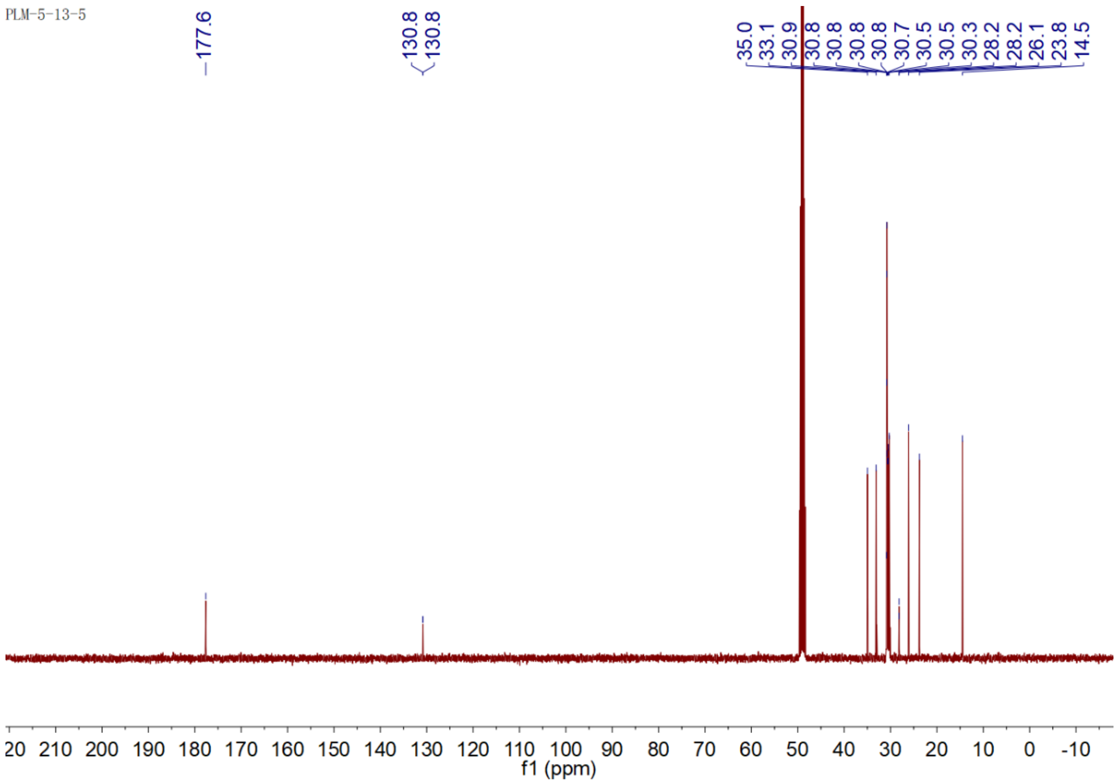

Figure S62 ^{13}C-NMR spectrum of compound **13**.
